# Contemporary opportunities and potential of Auger electron-emitting theranostics

**DOI:** 10.7150/thno.124671

**Published:** 2026-01-14

**Authors:** Seok-Yong Lee, H. Charles Manning

**Affiliations:** 1 Department of Nuclear Medicine, The University of Texas MD Anderson Cancer Center, 1881 East Rd, Houston, TX 77054, USA.; 2 Cyclotron Radiochemistry Facility, The University of Texas MD Anderson Cancer Center, 1881 East Rd, Houston, TX 77054, USA.; 3 RADIATE Radiopharmaceutical Research and Development Platform, The University of Texas MD Anderson Cancer Center, 1881 East Rd, Houston, TX 77054, USA.

**Keywords:** theranostics, Auger electron-emitting radionuclide therapy, radiopharmaceuticals

## Abstract

Recent breakthroughs in radiopharmaceutical (RP) therapy have emerged interest in employing Auger electron (AE)-emitting radionuclides as potential agents for precise theranostics. AE provides energy with exceptional localization due to their short tissue penetration range (TPR, < 10 nm), rendering them particularly effective for targeting nuclear DNA in tumor cells. In this context, AE-emitting radionuclide therapy (AE-emitting RLT) enables the targeted destruction of tumor cells while reducing harm to adjacent healthy tissue, a significant challenge in this field. Preclinical and early clinical investigations reveal the efficacy of AE-emitting RLTs in the theranostics of diverse malignancies, such as glioblastoma, prostate cancer, and neuroendocrine tumors. Notwithstanding these developments, challenges and limitations persist regarding dosimetry, delivery efficiency, and the treatment of radiotoxicity. A new paradigm is being developed to tackle the obstacles encountered by integrating molecular target markers (e.g., PARP) that function near the nucleus to improve the intranuclear delivery efficiency of AE-emitting radionuclides. Novel radiochemical methods such as these have facilitated the more stable and efficient labeling of biomolecules with AE-emitting radionuclides. Also, recent advances in DNA-molecular targeting, nanoparticles, nucleic acid/protein engineering, click- or bioorthogonal conjugation chemistry, and artificial intelligence (AI)-based structure modeling present concrete opportunities to overcome these limitations. Moreover, the integration of diagnostic imaging companion platforms employing theranostic radioisotope pairings facilitates real-time assessment of therapeutic efficacy and biodistribution, resulting in the formulation of enhanced treatment regimens. This review summarizes the prior development, recent advancements, barriers in clinical implementation, and future perspective of AE-emitting RLTs.

## 1. Introduction to Theranostics

**Figure 1:**
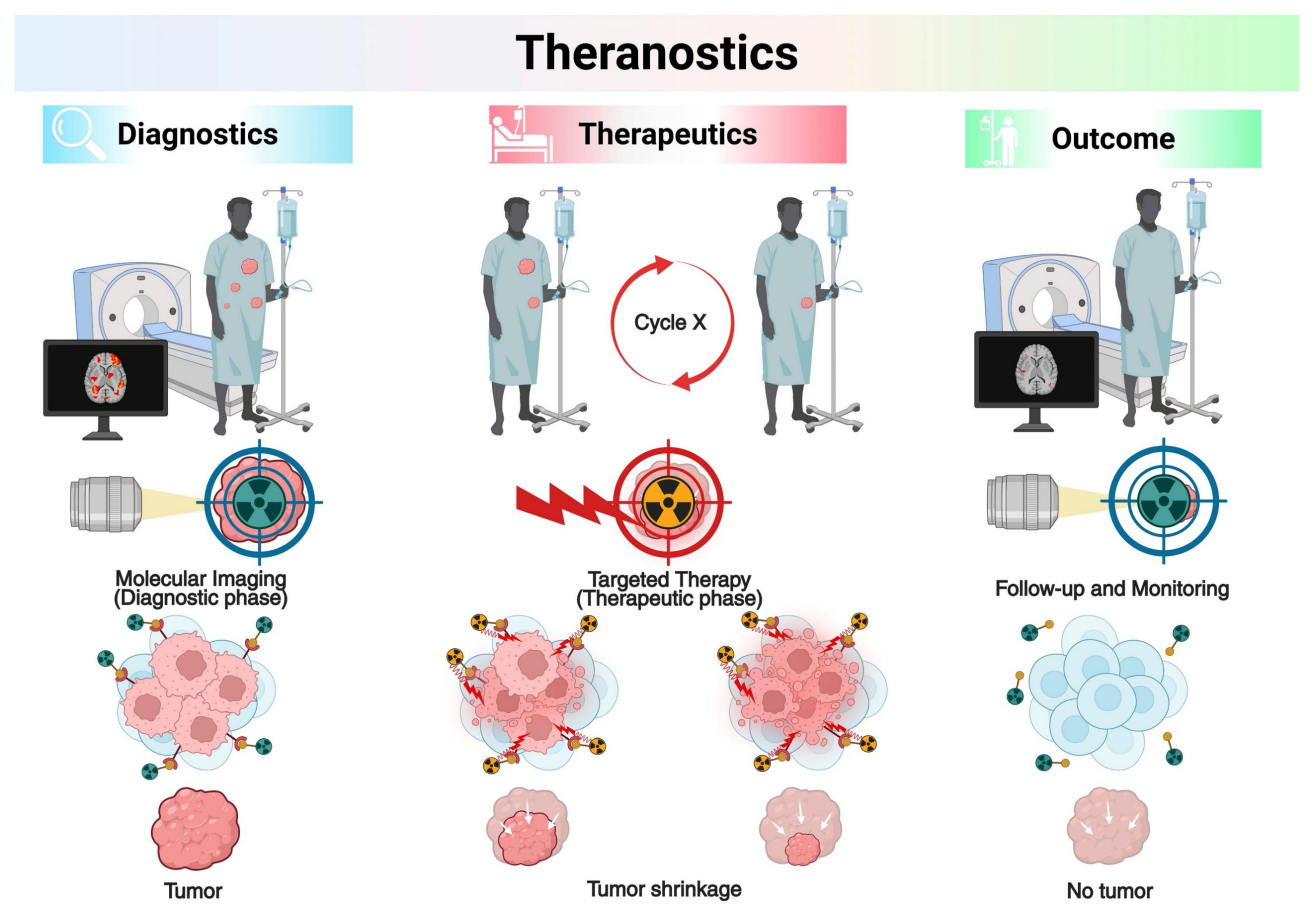
Illustration of theranostics with radiopharmaceuticals (RPs) pairs. Created with BioRender.com.

Theranostics represents an evolving paradigm in precision medicine that integrates diagnostic imaging and radioligand therapy (RLT), also known as targeted radionuclide therapy (TRT), using matched pairs of radiopharmaceuticals (RPs) labeled with diagnostic and therapeutic radionuclides 1, 2. This conceptual framework facilitates the validation of the existence and condition of biological targets via molecular imaging, followed by therapeutic intervention based on those findings 3. The primary function of theranostics is to identify candidates for RLT through imaging of the identical molecular target 4. The selection of patients usually includes clinically validated indicators and molecular biomarkers found in the target tissues. Theranostic strategies differ from traditional methods by first administering a radiolabeled diagnostic agent, enabling *in vivo* visualization of target expression through supplementary imaging modalities, followed by therapeutic intervention utilizing a chemically compatible compound labeled with a therapeutic radionuclide 5. TRT uses diagnostic radionuclides that give off gamma (γ-) photons or positrons (β^+^) to show where molecular targets are in real time within pathological lesions. Therapeutic radionuclides that emit alpha (α-), beta (β-), or Auger electrons (AE) can be used as substitutes for these diagnostic radionuclides to deliver targeted cytotoxic effects 6, 7. This diagnostic-therapeutic capability not only identifies patients likely to benefit from treatment before therapy but also allows for quantitative assessment of biodistribution, supports personalized treatment planning, and facilitates post-treatment dosimetric evaluation 8, 9. The combination of targeted RPs with other treatments, like external beam radiotherapy (EBRT) or immune checkpoint inhibitors (ICIs), has made it possible to treat patients with advanced or treatment-resistant cancers in new ways. The key point is regulatory milestones have accelerated clinical translation: [^177^Lu]Lu-DOTATATE (Lutathera^®^, Novartis) was approved by the U.S. Food and Drug Administration (FDA) on January 26, 2018, for somatostatin receptor-positive gastroenteropancreatic neuroendocrine tumors (GEP-NETs) 10, 11, [^68^Ga]Ga-PSMA-11 (Locametz^®^, Novartis) was approved on December 1, 2020, for prostate-specific membrane antigen (PSMA)-positive prostate cancer imaging 12, 13; and [^177^Lu]Lu-PSMA-617 (Pluvicto^®^, Novartis) was approved on March 23, 2022, for PSMA-positive metastatic castration-resistant prostate cancer (mCRPC) 14, 15. The latter has established itself as the first blockbuster RP in the history of nuclear medicine. These milestones highlight the capacity of theranostics to provide personalized medicine by individualizing the therapeutic index for each patient, achieving an exact equilibrium between efficacy and safety (**Figure 1**).

The β-emitting radionuclide [^177^Lu]Lu- possesses favorable physical/chemical properties that have contributed to its broad clinical use; however, these same features can also induce off-target organ and bone marrow toxicity, ultimately restricting the amount of radioactivity that can be administered safely 16. **Section 2** elaborates extensively on the physical features of therapeutic radionuclides. Conversely, researchers are investigating α-emitting radionuclides for application in several cancer types, including mCRPC, due to their ability to selectively eradicate tumor cells while minimizing damage to adjacent healthy tissues 16. AE-emitting radionuclides offer an added advantage: they demonstrate enhanced focused cytotoxicity at the single-cell level and greater localized energy relative to β-emitting radionuclides. These properties position AEs as a promising option for malignancies that are refractory to other treatments or for metastatic disease with a microscopic tumor burden 17. Although numerous preclinical studies have demonstrated the potent biological effect/efficacy of AEs, ongoing early-phase (phase I/II) clinical trials remain preliminary yet noteworthy 18-22. To date, however, no AE-emitting RLT has achieved widespread clinical adoption.

We summarize the therapeutic mechanisms, including microdosimetry, of widely used β- and α-emitting radionuclides alongside AE-emitting radionuclides. We also provide the current chemical limitations preventing widespread realization of AE-emitting RLTs and various potential mitigation strategies to overcome them. We critically present prior development efforts, including both successful and failed studies, to highlight barriers hindering clinical translation. Furthermore, this review overviews existing and promising suitable/potential pairs of AE-emitting radionuclides for companion diagnostics imaging and suggests a perspective for the future of AE-emitting RLTs.

## 2. Mechanism of Therapeutic Radionuclides in Nuclear Medicine

**Figure 2:**
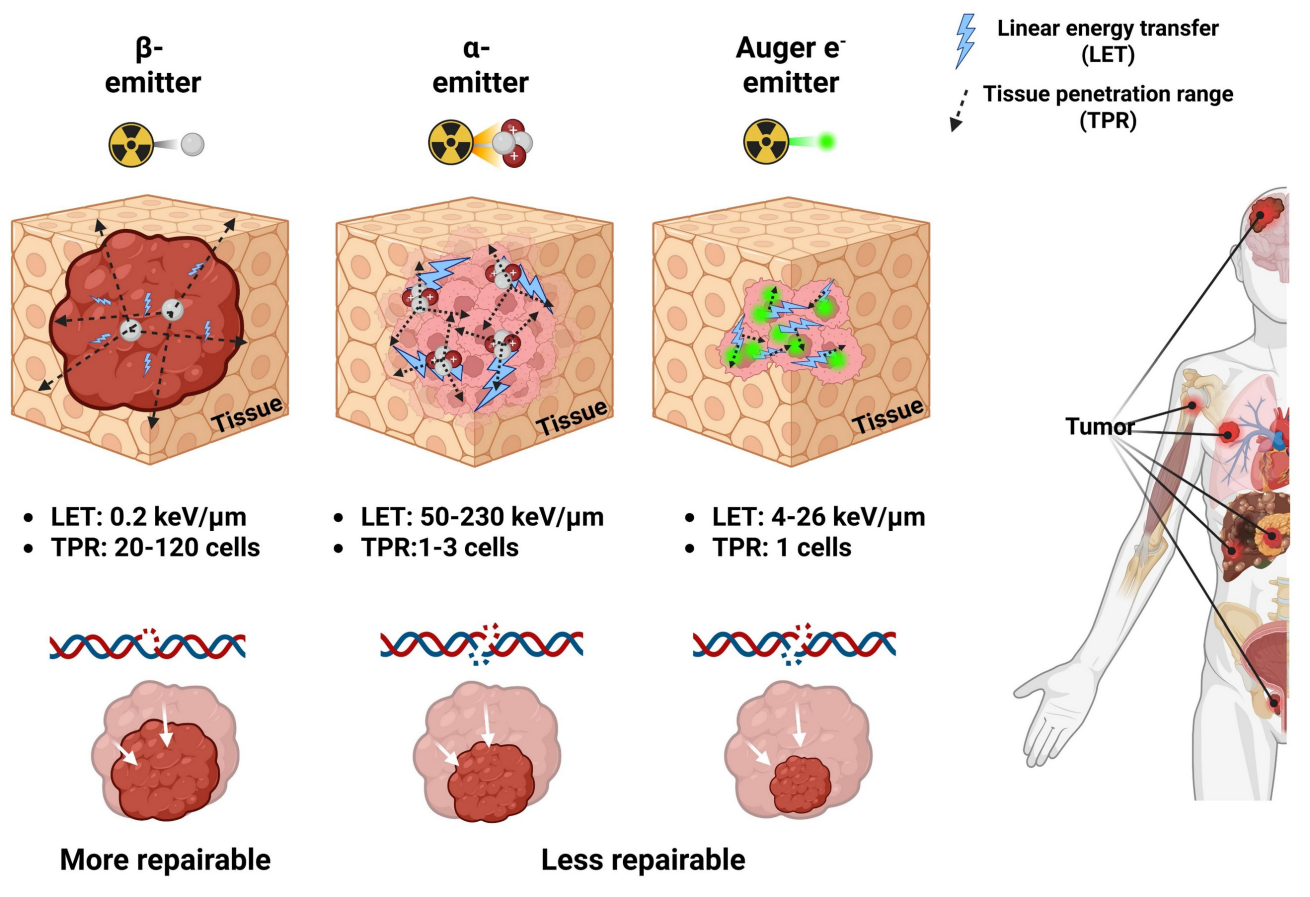
Schematic comparison of properties of β-, α-, and AE-emitting RLTs. Low LET radiation from β-emitting radionuclides primarily causes DNA single-strand breaks (DNA SSBs), which are repairable but can result in cell death when repair fails by DNA repair systems. The high TPR of β-particles increases the risk of off-target toxicity in nearby normal tissues and bone marrow. Conversely, α- and AE-emitting radionuclides deliver high LET radiation that induces irreparable DNA double-strand breaks (DNA DSBs), and their shorter TPR enables highly localized single cell levels cytotoxicity with minimal injury to adjacent healthy tissues. Created with BioRender.com.

Depending on the emission characteristics of the radionuclide, RPs may serve either diagnostic or therapeutic purposes. Photon emissions such as X-rays, γ-rays, and positrons are primarily employed for imaging and target verification, whereas β-particles, α-particles, and AE (**Figure 2**) mediate therapeutic effects. A clear understanding of the nature of each type of radiation and its corresponding biological mechanism is critical, as the selection of an appropriate therapeutic approach may vary significantly depending on these factors.

### 2.1 β-particles

β-particle emission is a type of radioactive decay that occurs in nuclei with an excess of neutrons over protons. During this process, a neutron is converted into a proton, releasing a high-energy electron, a β-particle, and an antineutrino 23. These β-particles have tissue penetration ranges (TPR) that are relatively long, commonly involving 0.5 and 12 mm (the same as the diameter of about 20 to 120 cells). They also emit low linear energy transfer (LET) radiation with values between 0.1 and 1.0 keV/μm 24. This property allows β-emitting radionuclides to induce cytotoxicity within tumors by generating reactive oxygen species (ROS) and causing predominant DNA single-strand breaks (DNA SSBs) 25. Such DNA damage is relatively minor compared with the DNA double-strand breaks (DNA DSBs) induced by α-/or AE-emitting radionuclides, which release higher linear energy. DNA damage caused by the low LET of β-emitting radionuclides is more amenable to repair, but if the repair process fails, it may ultimately lead to apoptotic cell death (**Figure 2**). β-emitting radionuclides are especially beneficial for treating tumors with different types of target expression due to the "crossfire-effect." It occurs when radiation from targeted cells extends to adjacent non-targeted or weakly expressing cells, rendering treatment more effective in solid tumors 26, 27. [^90^Y]Y-, [^131^I]-, and [^177^Lu]Lu- belong to several β-emitting radionuclides that have been approved for clinical use 28. In particular, the FDA-approved RPs Lutathera^®^ and Pluvicto^®^ utilize [^177^Lu]Lu- to deliver TRT 29, 30. [^177^Lu]Lu-, with a half-life of 6.7 days, emits γ-photons at 113 keV and 208 keV, enabling high-quality single photon emission computed tomography (SPECT) imaging alongside β-particles with a maximum energy of 497 keV, making it especially well-suited for theranostics applications 31. However, its relatively low LET and longer TPR may increase the risk of damaging adjacent healthy tissues. In this context, α-emitting radionuclides have attracted interest in their ability to deliver highly localized, powerful cytotoxic effects with low off-target toxicity, especially in the treatment of small-volume or micro-metastatic illness, including inside bone marrow compartments 32.

### 2.2 α-particles

Radionuclides that undergo α-decay emit out α-particles, which are composed of two protons and two neutrons (helium-4 nuclei). These particles have high LET values between 50 and 230 keV/μm. Even though their TPR ranges from 20 to 100 μm, which is only 1 to 3 cell diameters, the high LET makes it possible for an immense amount of energy to be deposited along the particle path 33, 34. This results in complex and often irreparable DNA DSBs, contributing to pronounced cytotoxic effects compared to β-emitting radionuclides (**Figure 2**) 35, 36. α-emitting RLT is especially advantageous in the treatment of micrometastatic lesions or hematologic malignancies, where radiation can be confined to targeted cells with minimal exposure to adjacent normal tissue 37, 38. The primary α-emitting radionuclides being studied are [^211^At]At-, [^212^Bi]Bi-, [^212^Pb]Pb-, [^213^Bi]Bi-, [^223^Ra]Ra-, [^225^Ac]Ac-, and [^227^Th]Th- 39. Of the above, [^223^Ra]RaCl₂ (Xofigo^®^) is the only FDA-approved treatment. It was approved in 2013 for mCRPC with bone involvement 40, 41. Novel therapies, including [^225^Ac]Ac-PSMA-617, have shown much promise in phase I clinical trials (AcTION, NCT04597411). Approximately fifty percent of the patients with mCRPC who received the therapy had a clinically significant drop in their prostate-specific antigen (PSA) levels, and early data suggest that it may also improve overall survival. [^225^Ac]Ac-PSMA-617 may be an improvement over β-emitting radionuclide-based treatments like [^177^Lu]Lu-PSMA-617 (Pluvicto^®^) 42. Nonetheless, the production of α-emitting radionuclides remains constrained by high costs and a lack of infrastructure for making them 43. Moreover, the recoil of daughter isotopes during decay cascades may result in systemic redistribution, leading to possible toxicities in non-target organs 44, 45. These limitations necessitate innovative solutions in radionuclide production, chelator development, and biological containment of decay progeny.

### 2.3 Auger Electrons

**Figure 3:**
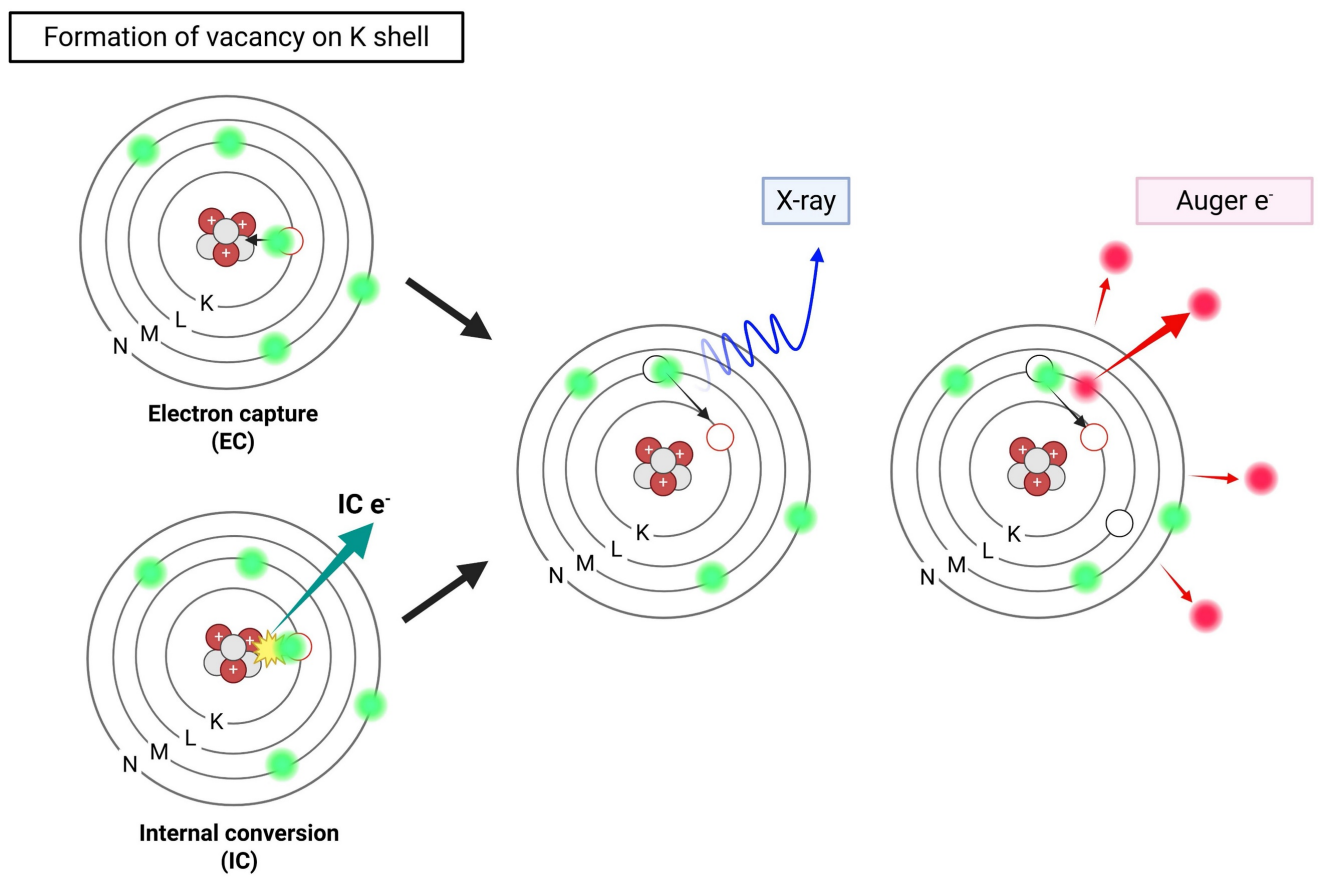
Illustration of Auger electron-emitting radionuclides. Created with BioRender.com.

Certain radionuclides often employed in nuclear medicine imaging undergo decay via electron capture (EC) or internal conversion (IC), resulting in the emission of a cascade of low-energy electrons known as AEs. In EC, a proton-rich nucleus assimilates an inner-shell electron, often from the K shell. This changes a proton into a neutron and leaves a vacant space in the electron orbital. As an electron from a higher energy shell (e.g., L shell) fills this vacancy, excess energy is released either as a characteristic X-ray or transferred to another electron, which is then ejected from the atom—this ejected electron is termed an AE 46. In parallel, IC occurs when an excited nucleus de-excites by transferring its excess energy directly to an orbital electron, ejecting it from the atom instead of emitting a γ-photon (**Figure 3**) 47. Most AEs possess relatively low energies, typically below 26 keV, with a maximum reported at 78.2 keV (e.g., from [^195^Pt]Pt-), but exhibit extremely short TPR, often under 0.5 µm. This behavior corresponds to a high LET ranging between 4 and 26 keV/μm, significantly exceeding that of β-particles 48, 49.

The dense ionization cascade generated by AEs induces high LET-type radiotoxicity through complex molecular alteration, including lipid peroxidation and protein oxidation. When AEs are emitted near the nuclear DNA, particularly within the cell nucleus, they can cause irreparable DNA DSBs (**Figure 2**) 50, 51. Unlike β-emitting radionuclides, radiation types such as AEs and α-emitting radionuclides may be particularly suitable for eradicating single cells or small-volume disease (< 1 cm). The combination of high LET and ultrashort path length allows AE-emitting radionuclides to deliver precise cytotoxic effects with minimal off-target damage—making them mechanistically like α-emitting radionuclides 52, 53. However, unlike α-emitting radionuclides, AE-emitting radionuclides typically produce either stable or no radioactive daughter products, thereby reducing concerns related to systemic redistribution and non-target toxicity 45, 46. Notably, [^111^In]In-, [^99m^Tc]Tc-, [^67^Ga]Ga-, [^125^I]-, and [^201^Tl]Tl- also co-emit γ-photons, enabling their dual use for theranostics by providing both therapeutic AE and γ-emissions suitable for SPECT or γ-scintigraphy, thus offering robust tracking and administered activity verification capabilities in preclinical and clinical settings (**Table 1**) 54-58.

### 2.4 Microdosimetric Comparison of β-, α-, and AE-emitting Radionuclides

Despite macroscopic characteristics such as particle energy, LET, and TPR being crucial for comprehending the functionality of emitters, they do not entirely elucidate the varying biological effects observed in TRT. Increasing evidence indicates that the spatial pattern of energy deposition at micrometer- and nanometer-scale dimensions plays an equally critical role in defining therapeutic efficacy and normal tissue toxicity. To align these physical characteristics with biological response and translational impact, a microdosimetric framework is necessary. Therefore, in this section, we review the emitter-specific microdosimetric signatures of β-, α-, and AE-emitting radionuclides and discuss how these nanoscale dose distributions shape their therapeutic windows and vector design considerations 59-61.

β-emitting radionuclides produce low-frequency lineal energy (γ) spectra, with energy distribution below 1 keV/µm, reflecting primarily long-range, sparsely ionizing track structures 62-64. Stochastic microdosimetry simulations using TOPAS-nBio and Geant4-DNA support that β-particles primarily induce isolated SSBs with a low probability of forming complex DNA damage clusters 65-68. This extensive and diffuse dose distribution can be advantageous for treating large or diffuse tumors, but its efficacy is limited for treating micro-scale lesions 69. In contrast, α-emitting radionuclides exhibit a fine dose distribution spectra in the range of 50-200 keV/µm, characterized by high-density linear track structures and minimal lateral scattering 70, 71. High-resolution measurements using tissue-equivalent proportional counters (TEPC) and nanodosimetric chambers have demonstrated that α-particles generate high-density ionization clusters along their tracks, including complex DSBs that are difficult to repair 72, 73. At the clinical level, these microdosimetric properties account for the potent cytotoxicity of α-emitting radionuclides against micrometastases and small-volume tumors, while also highlighting the risk of off-target toxicity if targeting vectors are not sufficiently specific 74, 75.

AE-emitting radionuclides exhibit fundamentally distinct and highly localized microdosimetric distributions. Following decay, tens to hundreds of low-energy electrons are emitted in rapid cascades, generating extremely high local energy within a few nanometers of the decay site (absorbed doses per-decay are on the order of tens to hundreds of kGy) 76, 77. These confined ionization clusters induce irreparable DNA DSBs, offering considerable therapeutic potential, which can only be harnessed when controlled to precisely reach DNA beyond intracellular migration 78. Track-structure modeling at the scale of DNA base pairs indicates that the relative biological effectiveness (RBE) of AE-emitting radionuclides increases rapidly with nuclear or perinuclear localization, exceeding even α-emitting radionuclides on a per-decay basis when decay occurs in the direct vicinity of DNA 79, 80. Microdosimetric assessment at cellular and subcellular tiers can be performed by computing dose point kernels and S-values using general-purpose electron/photon Monte Carlo software such as PENELOPE/PenEasy 79, 81. Nevertheless, due to the radiological nature of AE-emitting radionuclides, it is recommended to utilize specific nanodosimetry measurement codes such as Geant4-DNA or TOPAS-nBio for a comprehensive understanding of the tracheostomy structure, cluster formation, and chemical effects at the DNA level.

Microdosimetry offers criteria for the selection of radionuclides appropriate for clinical translation. As next-generation AE-emitting RLTs and potential mitigation strategies advance through the translation pipeline, the incorporation of microdosimetry parameters in preclinical evaluation and treatment planning is increasingly highlighted. The table below summarizes and compares Monte Carlo-based microdosimetry information for therapeutic radionuclides, including α-, β-, and AE-emitting radionuclides (**Table 2**).

## 3. Emerging Auger Electron-emitting Radionuclides: Current Limitations and Potential Mitigation Approaches

### 3.1 Key Challenges and Limitations

AE-emitting radionuclides offer unique opportunities for precision cancer therapy due to their highly localized and potent radiobiological effects. The therapeutic potential of radionuclides emitting AE can theoretically be determined by the average number of AEs emitted per nuclear decay. Radionuclides with higher AEs emissions can also enhance their therapeutic effects with lower doses due to the lower number of decays requiring irreversible DNA damage. Therefore, AE-emitting radionuclides ([^195m^Pt]Pt-(36.5 AE/decay), [^193m^Pt]Pt-(27.4), [^119^Sb]Sb-(23.7), [^125^I]-(23.0), and [^201^Tl]Tl-(20.9)) are generally preferred in TRT. Conversely, commonly used isotopes such as [^123^I] (13.7), [^161^Tb]Tb- (10.9), [^111^In]In- (7.4), [^67^Ga]Ga- (4.9), [^99m^Tc]Tc- (4.4), and [^64^Cu]Cu- (1.8) have significantly lower incidence of side effects, which may require increasing the activity of radioactivity administered or extending the residual time in the body to achieve similar cytotoxic effects 47. Nevertheless, this may be the basis for prioritizing radionuclides that produce high-yield AE, which presupposes that the AE-emitting radionuclides penetrate sufficiently into intracellular DNA and their effectiveness is verified. This is because if radionuclides are confined to cytoplasm or cell surface, their therapeutic effects may be limited. Additionally, it can be argued that enough care should be given to whether factors like half-life may enhance therapeutic efficacy and whether the development and supply of high-yield AE-emitting radionuclides can fulfill demand.

Although AE-emitting radionuclides can deliver highly localized and potent cytotoxic effects, truly AE-pure isotopes are rare, as most also emit high-energy β-particles or conversion electrons (CEs) alongside diagnostic X- and γ-rays. These unintended emissions can reduce the primary therapeutic advantage of AE radionuclides, which is precise targeting with minimal collateral damage. Consequently, the photon-to-electron (p/e) energy yield ratio has become an important ancillary criterion in the clinical evaluation of AE-emitting RLTs 82. Among the most promising AE-emitting radionuclides are the platinum-based isotopes [^195m^Pt]Pt- and [^193m^Pt]Pt-, which display remarkably high AE yields per decay and emit low-energy γ-photons (98.9 and 66.8 keV, respectively), leading to low p/e energy ratios of 0.42 and 0.09 82. Although moderate γ-emission might permit valuable imaging and dosimetry applications, excessive γ-radiation as observed with isotopes such as [^125^I]-, [^123^I]-, [^161^Tb]Tb-, [^111^In]In-, [^67^Ga]Ga-, [^99m^Tc]Tc-, and [^64^Cu]Cu- may lead to unintended toxicity at therapeutic levels 83. Furthermore, because AE radiation has an inherently limited TPR and lacks a crossfire-effect, treating tumors with heterogeneous uptake patterns often requires high administered activities or repeated dosing, which can inevitably result in accompanying γ-photon emission. This p/e energy ratio is a factor that should be carefully considered in the clinical translation process of AE-emitting RLT optimization to ensure both efficacy and safety for patients and operators.

Effective application of AE-emitting radionuclides requires careful consideration not only of their radiophysical properties but also of limitations in chemical compatibility, nuclear reaction feasibility, and global availability. More than 65% of AE-emitting radionuclides identified to date are either unavailable or subject to severe supply restrictions, and their large-scale synthesis requires rare high-flux neutron reactors or high-energy α-particle cyclotrons, which are not widely accessible. An indirect technique for producing [^195m^Pt]Pt- via a double-neutron capture process on [^193^Ir]Ir- has been attempted; however, it suffers from low yields and challenges in purifying the chemically resistant [^193^Ir]Ir target material. Furthermore, isotopic contaminants such as [^192/194^Pt]Pt- can be produced during irradiation alongside [^195m^Pt]Pt-, necessitating complex chemical separation and purification strategies 84. High-purity [^193m^Pt]Pt- is often produced by the ^192^Os(α,3n) reactions or alternative high-energy irradiation techniques, which necessitate costly osmium targets and specialized accelerator equipment 85. Additionally, AE-emitting actinides such as [^231^Th]Th-, [^237^U]-, and [^239^Np]Np- exhibit excellent AE yields, but their clinical use is constrained by regulatory restrictions on nuclear material handling, complex decay chains, and difficulties in achieving acceptable purity levels. Despite their potent theranostic profile, as detailed in the following section, the clinical translation of AE-emitting RLTs using [^161^Tb]Tb- is fundamentally limited by constraints in its specialized supply chain and manufacturing scalability. No-carrier-added, high-specific-activity [^161^Tb]Tb- is primarily produced by neutron irradiation of highly enriched [^160^Gd]Gd- targets via the ^160^Gd(n,γ)^161^Gd → ^161^Tb reaction, representing the current standard for clinical-grade radionuclide production 86. However, significant hurdles are the limited availability of highly enriched [^160^Gd]Gd- target material and the rigorous, time-intensive radiochemical separation process necessary to obtain high-purity [^161^Tb]Tb- from neighboring lanthanide contaminants 87, 88. Isolating [^161^Tb]Tb- from the ^161^Dy generated concurrently with the predominant Gd target material is a challenging endeavor due to the closely analogous chemical characteristics of these lanthanide elements, necessitating meticulously refined techniques to attain high purity 89. Recent advances, including novel resin-based chromatographic techniques (e.g., P350@resin) 90, electrochemical oxidation 89, and integration into automated synthesis modules 91, 92, have enabled baseline-level separation within a few hours, supporting rapid and reproducible production suitable for clinical applications.

To develop targeted AE-emitting RLT, researchers should create stable radiometal complexes that exhibit compatibility with biological systems. The majority of AE-emitting radionuclides are metallic ions; therefore, effective bifunctional chelators are essential to ensure their strong adherence to the targeted vector and optimal functionality 93. Although thermodynamic stability, typically quantified by formation constants (K_ML_ = [ML]/[M][L]), indicates the strength of the binding between two molecules. However, kinetic stability under normal conditions is what usually determines whether a drug is safe to use in the clinic 94. Biological environments comprise several competing elements, such as endogenous metals, natural chelators, and reducing agents, which may compromise the stability of metal complexes. Extensive research has been conducted on chelators such as 1,4,7,10-tetraazacyclododecane-1,4,7,10-tetraacetic acid (DOTA), diethylenetriaminepentaacetic acid (DTPA), 1,4,7-triazacyclononane-1,4,7-triacetic acid (NOTA), and ethylenediaminetetraacetic acid (EDTA) for lanthanides that emit AE; however, further investigation is required to explore lesser-studied isotopes and their coordination properties 94. The reversible oxidation-reduction dynamics between Sb(III) and Sb(V) of [^119^Sb]Sb, along with its intricate coordination chemistry involving oxygen, sulfur, and nitrogen donors, complicate the development of a stable and long-lasting chelator for use in the body 95. DOTA and NOTA, prevalent macrocyclic ligands, lack sufficient stability *in vivo*. This prompts concerns regarding chelate dissociation, off-target effects, and the reduced effective dosage at the tumor location 96, 97. Recent studies on chelator chemistry using Sb(V)-based coordination techniques show promise; however, these developments remain in the preclinical phase, and clinical implementation has not yet occurred 98, 99. The effective production of AE-emitting RPs hinges on the selection of an appropriate chelator. This involves investigating factors like stability, mobility, and efficacy in cancer targeting.

AE-emitting radionuclides exhibit exceptionally high cytotoxicity when decays occur near DNA, a strategy that has historically produced potent DNA DSBs and strong cell kill compared with cytoplasmic localization. However, this advantage can have drawbacks, as non-specific binding or intercalation of AE-emitting constructs into DNA of non-tumor proliferating cells may result in off-target DNA damage, thereby narrowing the therapeutic window 100. Indeed, radioconjugates containing DNA-intercalating moieties (e.g., an acridine-orange group linked to a radionuclide) have demonstrated that the RBE of AEs strongly depends on proximity to DNA; when the radionuclide is not sufficiently close to the double helix, the yield of DSBs and cytotoxic effects decreases dramatically 101. Furthermore, recent high-resolution, simulation-based nanoscale dosimetry studies indicate that energy deposition and DNA break yields from [^125^I] decay are highly sensitive to the precise positioning of AE-emitting radionuclides relative to individual base pairs 59, 102. This implies that even minor deviations in subcellular or local geometry, such as imperfect nuclear import or non-uniform chromatin structure, can shift AE decays outside the critical nanometer range, thereby reducing efficacy or, conversely, causing unpredictable off-target genotoxicity. Thus, it may be inferred that dosimetry models at both macroscopic and cellular levels in AE-emitting RLTs are inadequate for consistently ensuring safety or predicting treatment outcomes 59.

AE-emitting radionuclides can directly induce DNA DSBs through concentrated interactions or indirectly cause DNA damage through the generation of ROS. If this damage is not repaired, it can lead to cell death. However, misrepair can result in chromosomal abnormalities, micronuclei formation, and other characteristics of genomic instability. For example, a study using [^123^I]IUdR in human lymphocytes reported a dose-dependent increase in micronuclei formation even at low doses (~0.15 Gy) using the cytokinesis-block micronucleus assay, and the RBE values were relatively high, ranging from 3 to a maximum of 10 compared to γ-radiation 103. While many preclinical studies report chromosomal abnormalities or cytotoxicity, the relationship between acute AE-induced DNA damage and the risk of transformation, including long-term genomic instability or persistent rearrangements and secondary malignancy development, is not well understood. Furthermore, heterogeneity in uptake, nuclear entry, chromatin binding, and cellular repair capabilities among different cell types (tumor cells *vs*. normal cells) leads to unpredictable chromosomal instability. It should also be considered that if AEs accumulate in the perinuclear region instead of directly binding to DNA, their accumulation could reduce efficacy and induce chromosomal instability in normal tissues.

The key limitations of AE-emitting radionuclides as successful RPs, along with their mechanistic/technical basis, impact on clinical translation, and potential mitigation strategies, are summarized in the table below (**Table 3**).

### 3.2 Potential Mitigation Strategies

#### 3.2.1 PARP

Poly(ADP-ribose) polymerase (PARP) is an important biomarker that is found in many different types of tumors. In cancer cells, the DNA SSB repair process involves PARP enzymes, which help change the structure of chromatin and bring in DNA repair factors that allow the stalled replication forks 104. Inhibiting PARP prevents the repair of DNA DSBs, leading to the accumulation of unresolved lesions that ultimately result in cell death. Utilizing this concept, PARP inhibitors have evolved into pharmaceuticals that have the potential to treat several different types of cancer, both on their own and with other treatments 105. Given that PARP1 functions near the nucleus, conjugating AE-emitting radionuclides to PARP-targeted ligands has the potential to deliver electron cascades within just a few nanometers of DNA, thereby maximizing therapeutic efficacy by depositing energy directly in nuclear and perinuclear regions. This data indicates that PARP1-targeted AE-emitting RLT may provide an effective approach to address the significant drawbacks of traditional delivery vehicles (e.g., antibodies or peptides), which frequently persist in the cell membrane or cytoplasm, leading to diminished nuclear delivery efficiency and increased targeting variability. Preclinical studies across five major tumor types, including glioblastoma multiforme (GBM) 106, triple-negative breast cancer (TNBC) 107, prostate cancer 108, ovarian cancer 109, and pancreatic cancer 110, strongly support the clinical applicability of PARP-targeted AE-emitting RLT. These results suggest that increased PARP1 expression, genomic instability, or defects in DNA repair processes represent a powerful molecular target for the application of AE-emitting RLT strategies that need to reach the DNA in various tumor types. Despite its broad applicability, the intrinsically short path length of AEs requires precise intranuclear delivery, which may be inconsistent in heterogeneous tumors or in those with limited PARP accessibility. The lipophilicity of the RPs, efflux mechanisms, suboptimal pharmacokinetics (PK), and variability in DNA-binding efficiency further limit dose deposition at the chromatin level. Currently, most of the available data remains in the preclinical stage, limiting clinical validation, and there may be considerable uncertainty regarding toxicity to normal tissues, particularly in proliferative tissues with endogenous PARP expression. Further optimization of biomarker-based patient selection is needed for application of AE-emitting RLTs targeting PARP 111.

#### 3.2.2 Nanoparticles

Nanoparticles (NPs) are widely applied as carriers for TRT, representing a promising strategy to overcome major challenges hindering clinical application by amplifying radioisotope payload, facilitating targeting and cellular uptake, improving PK, and enabling multifunctional design (e.g., combination with sensitizers). In particular, the integration of organic and inorganic nanostructures ultimately provides a versatile platform for amplifying DNA damage induced by AE-emitting radionuclides. A detailed overview of these nanoparticle-based strategies is presented in the table below, highlighting the mechanisms and advantages of each NP's application in AE-emitting RLTs, its unique limitations and challenges, and representative research examples (**Table 4**) 112.

From a theranostic perspective, nanostructures in AE-emitting RLT offer several advantages: (i) enhanced radionuclide payload for improved multimodal tumor imaging contrast and therapeutic efficacy, (ii) improved programmable PK properties extending intratumoral retention time, (iii) targeted modularity enabling ligand exchange and multivalent binding on the nanoparticle surface, and (iv) control over mechanism-based intracellular trafficking pathways, including nuclear translocation or lysosomal escape, which are crucial for radionuclide efficacy. Furthermore, high-Z NP materials can go beyond acting as sensitizers to external beam radiation, inducing photoelectron emission at the nanoparticle surface to amplify local energy deposition or enhanced permeability and retention (EPR) effect in tumors, providing advantages consistent with theranostic optimization. NP-based radionuclide-emitting RLT has the potential to become a next-generation RLT platform for optimizing theranostic strategies through ongoing multidisciplinary research and development aimed at increasing the possibility of clinical translation.

#### 3.2.3 Click Chemistry and Biorthogonal Radioconjugation

The short range from AE-emitting radionuclides makes their cytotoxicity critically dependent on the proximity of the radionuclide to the DNA, motivating research toward agents capable of efficient nuclear localization. The development of these sophisticated RPs is strongly supported by click chemistry radiolabeling, which allows the rapid, high-yielding, and modular synthesis of complex bioconjugates under mild conditions, essential for handling short-lived radionuclides 143. Current research is focused on optimizing two main bioorthogonal approaches. The first, direct radiolabeling, employs reactions such as strain-promoted azide-alkyne cycloaddition (SPAAC) or inverse electron-demand Diels-Alder (IEDDA) to attach AE-emitting radionuclides to targeting vectors (e.g., peptides, antibodies) with high selectivity 144. The second, and more strategically significant, approach is pretargeting, in which an unlabeled targeting molecule is first allowed to accumulate at the tumor site, followed by administration of a smaller, radiolabeled “click” partner 145. This sequential delivery markedly enhances the tumor-to-background ratio (TBR) while minimizing systemic toxicity by rapidly clearing unbound radiolabeled small molecules 146. These efficient radiolabeling techniques are employed to produce a broader spectrum of radiotracers, including positron emission tomography (PET) or SPECT agents, and complement conventional AE-emitting radionuclides, hence enhancing the theranostic arsenal 147. It is essential to continue developing more rapid click chemistry and bioorthogonal conjugation reactions that exhibit enhanced efficacy *in vivo* to optimize the therapeutic window and reinforce the synergy between AE-emitting RLTs and click- and bioorthogonal conjugation chemistry in personalized nuclear medicine.

#### 3.2.4 Protein Engineering Strategies

The fundamental limitation of AE-emitting RLTs is the required proximity of the radioisotope to the nucleus or DNA, which necessitates strategies beyond simple extracellular binding. While nuclear localization sequence (NLS) engineering has long been used to promote intranuclear shuttling, current protein engineering efforts are focused on overcoming systemic barriers such as poor PK and off-target toxicity 148. A major trend is the transition from bulky monoclonal antibodies to high-affinity, rapidly clearing scaffolds such as nanobodies (variable domain of heavy chain-only antibody, VHHs) and affibodies 149. These smaller formats offer superior tumor penetration and achieve high TBR at earlier time points, a feature that is particularly critical for AE-emitting radionuclides 150. Another significant strategy involves the engineering of PK extenders to optimize *in vivo* circulation time 151. This goal is typically achieved by fusing the targeting scaffold to human serum albumin or by incorporating specific albumin-binding domains (ABDs) to enable temporary “hitchhiking” 152. The incorporation of albumin can enhance the EPR effect, resulting in a significant increase in tumor accumulation and a substantial reduction in renal excretion 153. A protein engineering strategy that integrated an ABD with a HER2-targeted nanobody enhanced the systemic half-life of [^125^I], facilitating uniform distribution of the drug throughout the body, which is essential for substantial remission following a single dose in preclinical models 154. A contemporary strategy in tumor RLT involves the integration of the tumor microenvironment (TME) with protein engineering 153. Cathepsin B-sensitive GFLG or MMP-sensitive PLGLWA linkers, widely employed for spatially regulated drug release via tumor- or lysosome-specific protein therapy activation, may be suggested 155, 156. Incorporating these linkers prior to NLS ensures the protein carrier remains stable and concealed during its transit through the body. When cleaved in the TME, the NLS is exposed, facilitating the protein's entry into the nucleus. A protein engineering-based strategy, combined with and integrated by conventional NLS in AE-emitting RLT, provides an effective approach to improve intracellular delivery and increase accessibility to DNA molecules, resulting in a synergistic effect 148.

## 4. Historical Use and Prior Development (Success/Failure) of AE-emitting RLTs

Therapeutic applications of AE-emitting radionuclides have received increasing attention in the field of theranostics, alongside α-emitting radionuclides. Despite growing interest and promising outcomes from numerous preclinical and early-phase clinical investigations, no AE-emitting RP has yet been approved by the U.S. FDA. This section of the review summarizes the historical utilization and developmental efforts, both successful and unsuccessful, of AE-emitting RLTs (**Table 5**).

### 4.1 Iodine-125 /or 123

#### 4.1.1 ^125^I- or ^123^I-5-iodo-2-deoxyuridine

5-Iodo-2′-deoxyuridine (IUdR), radiolabeled with [^125^I] or [^123^I], is a thymidine analog that integrates into DNA during replication and has been studied for its AE-mediated cytotoxic effects. In preliminary research, Chan *et al.* demonstrated that [^125^I]IUdR significantly elevated DNA DSBs and clonogenic cell death in V79 cells compared to [^3^H]TdR and [^131^I]IUdR, necessitating merely 0.0037 Bq per cell to induce three DSBs within 1 h, highlighting the markedly high LET effect of AE 157. Sahu *et al.* reported the therapeutic efficacy of [^125^I]IUdR in a rat model of leptomeningeal metastases, where a single administration (18.5 GBq/head), daily injections for 5 days (3.7 GBq/day), and continuous infusion over 5 days (0.0185 GBq/h, totaling 18.5 GBq) significantly extended the median time to paralysis to 11, 12, and 15 days, respectively. Radioactivity was rapidly eliminated from all tissues, except for the thyroid and neoplastic cells proliferating in the spinal cord. This indicates that [^125^I]IUdR exerts a specific anticancer effect in the treatment of leptomeningeal illness 158. Macapinlac *et al.* observed that in all four patients, [^125/131^I] showed a biexponential decay pattern after hepatic artery infusion and continued to accumulate in the tumor. They also estimated that 15-50% of the tumor cells were in the S-phase, suggesting suitability for IUdR incorporation 159. In a mouse ovarian tumor (MOT) model, the AE-emitting radionuclide [^123^I]IUdR showed significant antitumor activity, improving mean survival and increasing absolute survival by 20% after 7 weeks following intraperitoneal (IP) administration. The observed survival benefit, even at the lowest administered activities, underscores the potent cytotoxicity of AE when effectively localized to tumor DNA 160. Clinical investigations followed, including a phase 0 study with intracisternal [^123^I]IUdR demonstrating selective tumor targeting with favorable safety. Mariani *et al.* investigated the effect of radiosensitization in patients with hepatic metastases from colorectal cancer by intra-arterially administering [^123^I]IUdR, followed by systemic treatment with 5-fluorouracil (500 mg/m^2^) and 1-folinic acid (250 mg/m^2^), known as inhibitors of thymidylate synthase. Upon re-administration of [^123^I]IUdR one week later, the mean tumor uptake increased significantly from 9.1% to 14.0% injected activity (IA), representing an average enhancement in early tumor uptake of 78% 161. These results collectively point to the promise of IUdR-based strategies in targeting proliferative tumor fractions, with administered activity timing and repetition as key variables.

#### 4.1.2 [^125^I]DCIBzL

**Figure 4:**
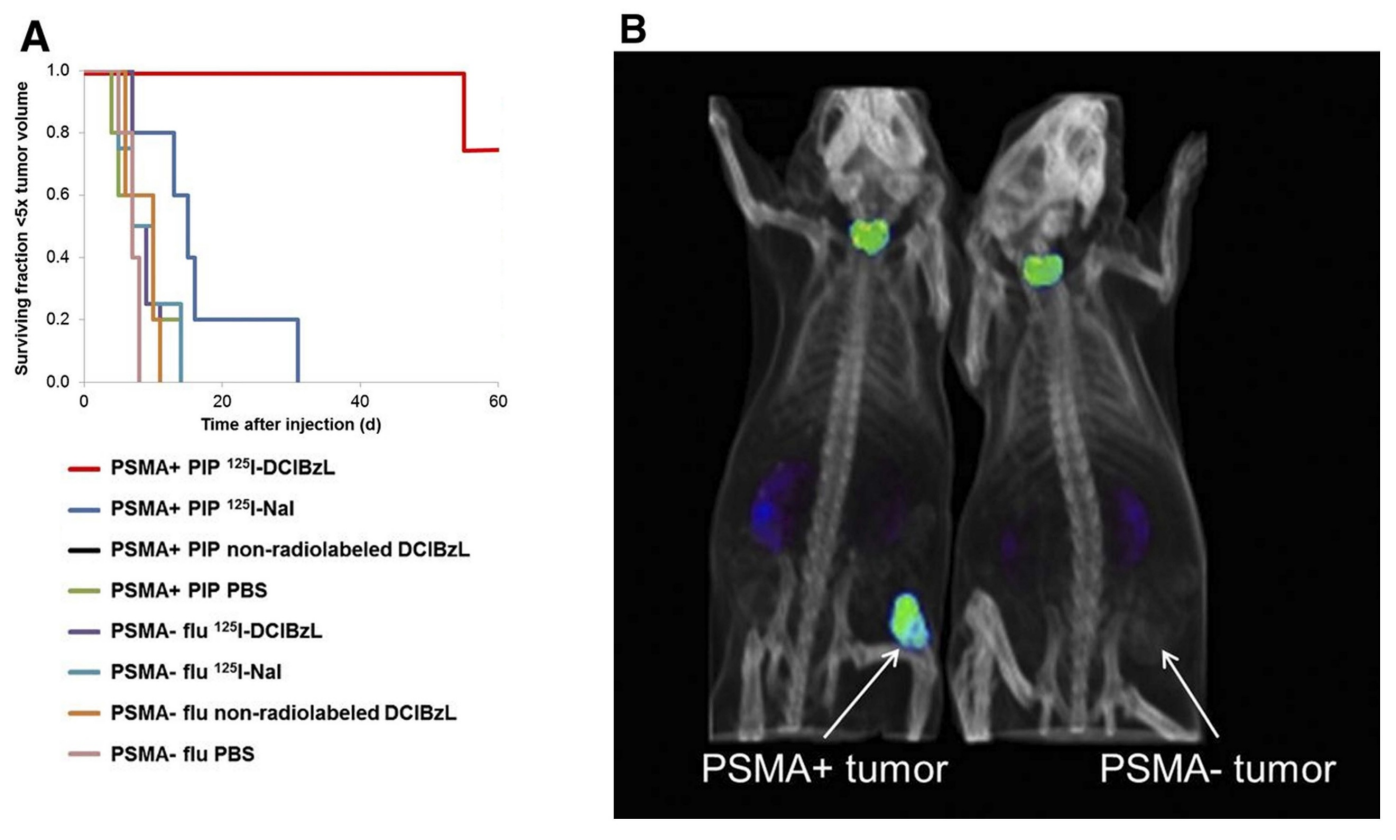
*In vivo* antitumor efficacy and SPECT/CT imaging with [^125^I]DCIBzL in tumor xenograft mouse models. (A) Tumor growth delay curve after treatment with 111 MBq of [^125^I]DCIBzL or an equal amount of control compounds. (B) Small-animal SPECT/CT maximum-intensity-projection (MIP) images acquired in two different tumor xenograft mouse models (PSMA-positive: PC3-PIP; PSMA-negative: PC3-Flu;) at 24 h after [^125^I]DCIBzL treatment. Copyright^©^ 2015 Society of Nuclear Medicine and Molecular Imaging.

2-3-[1-carboxy-5-(4-[^125^I]iodo-benzoylamino)-pentyl]-ureido]-pentanedioic acid ([^125^I]DCIBzL) targets PSMA, a type II transmembrane glycoprotein that is highly overexpressed in prostate cancer. Kiess *et al.* showed that [^125^I]DCIBzL treatment caused more DNA damage and less clonogenic survival in PC3-PIP (PSMA-positive) cells than in PC3-Flu (PSMA-negative) cells. Correspondingly, tumor growth in PSMA-positive xenografts was significantly delayed, with only one mouse reaching 5 times the initial tumor volume within 60 days, whereas the median time to this threshold in PSMA-negative and other treatment groups was less than 15 days (log-rank test, P = 0.002) (**Figure 4**) 162. Shen *et al.* showed that administering [^125^I]DCIBzL in activities between 18.5 and 111 MBq significantly delayed the appearance and growth of metastatic lesions in a micrometastatic prostate cancer model. The lesions manifested at a median of 4 weeks, in contrast to 2 weeks in the 0.37-3.7 MBq cohort 163. The median survival for mice receiving ≥ 18.5 MBq rose to 11 weeks, compared to 6 weeks for the control group. Notably, there was no significant toxicity observed even 112 days after treatment, based on changes in body weight, urinalysis, or necropsy results showing that the [^125^I]DCIBzL is safer than PSMA-targeted α-emitting radionuclides. Dosimetry modeling results confirmed that the dose absorbed by nuclei of kidney cells was significantly lower than the dose absorbed by tumor cell nuclei due to the limited range of AE emission and limited intracellular uptake, demonstrating the favorable therapeutic window of [^125^I]DCIBzL. However, despite these promising outcomes, no additional studies or clinical applications have been reported thus far.

#### 4.1.3 [^125^I]CLR1404

[18-(p-iodophenyl)octadecyl phosphocholine] is an alkyl-phosphocholine (APC) analogue that specifically targets lipid rafts. It preferentially infiltrates tumor cell membranes due to its affinity for microdomains abundant in sphingolipids and cholesterol. Grudzinski *et al.* demonstrated in a preclinical investigation that the ratio of absorbed dosage to tumors compared to absorbed radiation to bone marrow was favorable, with 0.261 Gy/MBq delivered to tumors and only 0.063 Gy/MBq to bone marrow. A single administration of 74 MBq [^125^I]CLR1404 enhanced the longevity of all treated animals to over 60 days and inhibited the growth of new triple-negative breast tumors by approximately 60% relative to controls. On day 35, lung metastases were markedly reduced. These results affirm the high therapeutic index and tumor specificity of CLR1404 as an AE-emitting radionuclide 164. Encouraged by these promising results, several phase I and II clinical trials have been initiated or completed to evaluate the theranostic potential of radiolabeled CLR1404 in various malignancies; however, these studies primarily employed the β-emitting radionuclide [^131^I], and investigations utilizing AE-emitting radionuclides remain lacking 185-187.

#### 4.1.4 [^125^I]35A7 mAb

**Figure 5:**
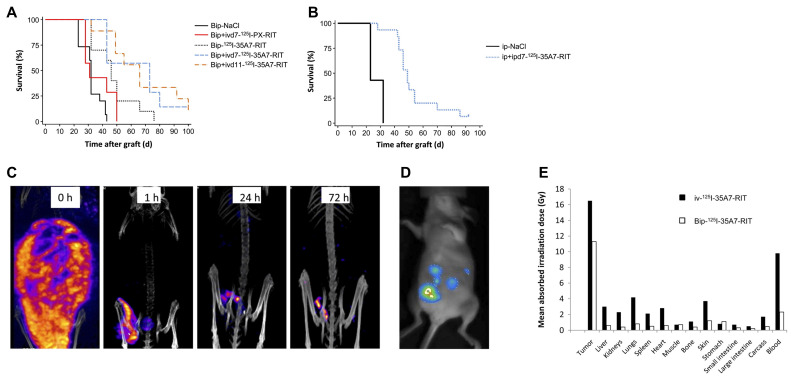
Intraperitoneal radioimmunotherapy (RIT) targeting small peritoneal carcinomatosis is conducted using high activities of the non-internalizing [^125^I]35A7. (A) Kaplan-Meier survival curves after administration of brief IP (Bip) or combined Bip and intravenous (IV) RIT in A431-bearing athymic nude mice model. (B) Kaplan-Meier survival rate after administration of Bip or combined Bip and IV 7-day RIT. (C) SPECT/CT images obtained immediately after injection (0 h) and 1, 24, and 72 h after lavage of the peritoneal cavity with saline in IP [^125^I]35A7 RIT (185 MBq) on day 4 post-implantation. (D) Bioluminescence images obtained 4 days post-implantation, immediately prior to RIT. (E) Graph of mean absorbed irradiation dose for brief IP [^125^I]35A7 RIT and IV [^125^I]35A7 RIT. Copyright 2010^©^ Society of Nuclear Medicine and Molecular Imaging.

Carcinoembryonic antigen (CEA) is a non-specific blood biomarker frequently raised in several malignancies, especially colorectal cancer and medullary thyroid carcinoma 188. It is widely used as a clinical indicator to monitor therapeutic response and detect recurrence in cancer patients 189. Targeting CEA with the non-internalizing monoclonal antibody 35A7 labeled with [^125^I]- has demonstrated promising antitumor efficacy in AE-emitting RLT by Santoro *et al. In vivo* investigations with A431 xenograft-bearing mice revealed that [^125^I]35A7-mAb produced a markedly higher tumor development delay and a 2.5-fold enhancement in median survival relative to its unlabeled variant, but internalizing [^125^I]m225 showed negligible differences in survival. Dosimetric analyses indicated a 7.4-fold higher tumor radiation administered activity for [^125^I]35A7 compared to [^125^I]m225, despite similar normal organ exposure. This is attributed to catabolite leakage following the internalization of m225, supporting the idea that nuclear localization is not a strict prerequisite for DNA damage induction 165. Boudousq *et al.* conducted brief intraperitoneal (Bip) radioimmunization by injecting 185 MBq (740 MBq/mg) of [^125^I]35A7 intraperitoneally into athymic nude mice on day 4 following peritoneal tumor xenograft. The peritoneal cavity was thoroughly rinsed with saline to remove unbound radioactivity after 1 h. Additional groups of mice received an intravenous (IV) injection of 37 MBq of [^125^I]35A7 either on day 7 or day 11 after xenografting, in combination with the Bip treatment. Control groups received either saline or irrelevant [^125^I]PX intravenously on day 7 following the short-term IP treatment. Mild and transient hematologic toxicity was observed after Bip radioimmunization and IV administration of [^125^I]-monoclonal antibodies, with no associated body weight loss. The median survival time increased from 32 days in the control group to 46 days in the short-term treatment group, 66 days in the group getting additional IV therapy on day 11, and 73 days in the group receiving IV therapy on day 7. The short-term treatment alone resulted in a threefold higher tumor-to-blood uptake ratio compared to standard IV treatment, with the mean absorbed tumor radiation dose being 11.6 Gy for the short-term therapy and 16.7 Gy with additional IV therapy. For healthy tissues excluding blood, the mean absorbed radiation dose did not exceed 1 Gy after short-term treatment and did not exceed 4.2 Gy following IV treatment (**Figure 5**) 166. Consistent with this, Piron *et al.* observed a time-dependent increase of γ-H2AX foci in both p53^+/+^ and p53^-/-^ HCT-116 cells subjected to low-activity [^125^I]35A7, indicating persistent DNA DSB. These findings endorse the investigation of alternate techniques utilizing non-internalizing vectors in AE-emitting RLTs 167.

#### 4.1.5 [^125^I]CO17-1A

CO17-1A is a thoroughly defined monoclonal antibody that specifically targets the epithelial cell adhesion molecule (EpCAM). Initially, it was derived from tumor cells obtained from individuals with colorectal cancer 190. Behr *et al.* evaluated the therapeutic efficacy, maximum tolerated activity (MTA), and maximum tolerated dose (MTD) of CO17-1A labeled with [^131^I]- and [^125^I]-. The MTA values for [^131^I]- and [^125^I]-labeled CO17-1A were 11.1 MBq and 111 MBq, respectively. Compared to unlabeled CO17-1A, [^131^I]CO17-1A induced a significant tumor growth delay of 7-8 weeks, after which exponential tumor regrowth occurred. In contrast, at equivalent toxic administered activities, [^125^I]CO17-1A demonstrated superior tumor growth suppression for up to 10 weeks, with approximately half of treated tumors exhibiting a partial remission characterized by a > 50% volume reduction. Beyond 10 weeks, tumor growth curves for [^125^I]CO17-1A showed a less steep slope than those of [^131^I]CO17-1A. Dosimetric analysis indicated that, considering electrons only, the blood absorbed dose was approximately 25% higher for [^125^I]- (21 Gy) than for [^131^I]- (17 Gy) at their respective MTDs. To assess whether the observed therapeutic advantage of [^125^I]CO17-1A was related to its internalization properties, control groups received 111 MBq of [^125^I]-labeled irrelevant anti-CD3 OKT3 antibody and a non-internalizing anti-CEA FC023C5 labeled with either [^131^I] (11 MBq) or [^125^I] (111 MBq). Similar marrow toxicity across groups confirmed equivalent dosing. The irrelevant OKT3 antibody demonstrated no tumor development delay compared to untreated controls, but the anti-CEA FC023C5 groups showed significant therapeutic benefit, irrespective of the radioisotope used. The tumor growth delay for [^125/131^I]-F023C5 was comparable to that of [^131^I]CO17-1A, suggesting that internalization may not be the sole determinant of treatment efficacy 168.

#### 4.1.6 [^125^I]mAB-425

Epidermal growth factor receptor (EGFR) is a recognized biomarker in high-grade gliomas, exhibiting overexpression in 60-90% of GBM patients, thereby presenting a potential therapeutic target 191. The anti-EGFR monoclonal antibody-425 (mAb-425), an IgG2a isotype generated by immunizing mice with A431 epithelial carcinoma cells, has been radiolabeled with iodine-125 ([^125^I]mAb-425) and has demonstrated direct anti-proliferative and anticancer effects in many preclinical experiments 192. Radiation-induced DNA damage exacerbates these effects, resulting in increased tumor destruction. Early phase I/II clinical trials administered adjunctive [^125^I]mAb-425 at administered activities of 18.5 GBq every three weeks, up to a cumulative administered activity of 5.18 GBq, in 180 patients diagnosed with GBM or anaplastic astrocytoma with dysplastic lesions 169, 170. The PK study indicated that the plasma half-life ranged from 18 to 24 h, whereas the tumor persisted in brain tissue for 48 to 72 h. The peak tumor absorption occurred approximately 16 ± 3 h post-injection. The median actuarial survival for GBM patients ranged from 4 to 15 months, but for anaplastic astrocytoma patients, it ranged from 4 to 270 months. Patients under 40 years of age with a Karnofsky performance status score of 70 or higher showed a significant survival advantage, with median survival times of 22.5 months and 65 months, respectively. Furthermore, a phase II trial by Li *et al.* involving 192 GBM patients administered IV [^125^I]mAb-425 at 1.8 GBq over three weeks (with a maximum cumulative activity of 5.4 GBq) showed a significantly prolonged median overall survival compared to a control group receiving standard therapy. The median survival was 12.1 ± 16.7 months in the monotherapy group (n = 97) and 14.9 ± 25.8 months in the temozolomide combination group (n = 51), in contrast to 8.4 ± 12 months in the control group (n = 39). Significantly, there was an absence of systemic toxicity or substantial damage to non-target organs. The immunogenicity arising from the murine components of the antibody and the resultant production of human anti-mouse antibodies limits the practicality of repeated administration. Utilizing humanized or chimeric anti-EGFR antibodies may enhance the efficacy and safety of the treatment 21.

### 4.2 Indium-111

#### 4.2.1 [^111^In]In-CO17-1A

Behr *et al.* investigate the biodistribution and therapeutic effects of radioimmunoconjugates labeled with various radionuclides, including [^125^I]CO17-1A, [^131^I]CO17-1A, and [^111^In]In-CO17-1A in the same animal model. Whole-body scans performed 48 h after injection showed little tumor uptake with the radioiodine-labeled antibodies, but clear tumor-specific signals were observed with the radiometal-labeled antibodies. To evaluate the therapeutic potential of β-emitting radionuclides, [^90^Y]Y- was employed alongside [^111^In]In-. The MTA and MTD were established at 4 MBq for [^90^Y]Y-CO17-1A and 85 MBq for [^111^In]In-CO17-1A. In a nude mouse xenograft model employing the human colorectal adenocarcinoma cell line GW-39, administration of [^111^In]In-CO17-1A resulted in a notable postponement of tumor growth at equitoxic levels relative to [^90^Y]Y-CO17-1A. This tumor suppression was comparable to that observed with the AE-emitting radionuclide [^125^I]CO17-1A at its MTD, which achieved complete remission without bone marrow transplantation. Dosimetry estimates, excluding photon contributions and considering only electron emissions, revealed similar blood doses of 24.8 Gy for [^111^In]In-CO17-1A and 24.2 Gy for [^125^I]CO17-1A at their respective MTDs 168. These findings suggest that monoclonal antibodies labeled with AE-emitting radionuclides such as [^125^I]- and [^111^In]In- may offer superior therapeutic efficacy over those labeled with conventional β-emitting radionuclides at equivalent toxic doses.

#### 4.2.2 [^111^In]In-DTPA-octreotide

Octreotide, a stable octapeptide analog of somatostatin, exhibits a high affinity for somatostatin receptor subtype-2 (SSTR-2), which is overexpressed in NETs 193. In 1991, octreotide was first labeled with the AE-emitting radionuclide [^111^In]In-conjugated to DTPA and employed for SSTR scintigraphy in tumor imaging 194. By 1994, [^111^In]In-DTPA-octreotide received U.S. FDA approval for the detection of primary localized and metastatic SSTR-2-positive neuroendocrine tumors 170. Krenning *et al.* reported a Phase I clinical trial in 30 patients with progressive SSTR-2-positive malignant NETs, administering up to 14 cycles of 6-7 GBq [^111^In]In-DTPA-octreotide with a maximum cumulative administered activity of 74 GBq. Among 21 patients receiving cumulative administered activity over 20 GBq, 8 achieved disease stabilization and 6 demonstrated tumor size reduction. Temporary decreases in platelet counts and lymphocyte subsets were observed in some patients, but clinical toxicities were minimal up to two years post-therapy 171. Meyers *et al.* reported that a 35-year-old patient with atypical carcinoid tumors, which were metastasizing to several locations, experienced disease stabilization for 14 months after receiving 8 biweekly cycles of 6.66 GBq [^111^In]In-DTPA-octreotide. Significantly, receptor-targeted RLT employing [^111^In]In-DTPA-octreotide offered a substitute for the gastrointestinal toxicity linked to chemotherapy 195. Valkema *et al.* conducted a Phase I trial in 50 patients receiving 2-11 GBq per cycle over 2 weeks to several months, with cumulative IA ranging from 20 to 120 GBq. Therapeutic responses were seen in 21 of 40 evaluable patients, including 14 with disease stabilization, 6 with minor remission, and 1 with partial remission. However, 3 of 6 patients receiving more than 100 GBq developed myelodysplastic syndrome or leukemia. Renal radiation absorbed doses per IA were approximately 45 Gy/100 GBq, twice the tolerance for external beam radiation, yet no nephrotoxicity was observed even at administered activity exceeding 100 GBq, demonstrating no harm of the short-range AE on kidney function 18. Limouris *et al.* administered intra-arterial [^111^In]In-DTPA-octreotide to 17 patients with unresectable SSTR-2-positive metastatic hepatic NETs, with a mean dosage of 6.3 ± 2.3 GBq. They demonstrated that 5.9% of patients achieved complete remission, 47% attained partial remission, and 17.7% experienced stabilization of their condition. In a cohort of 12 patients, the median overall survival was calculated to be 32 months, with 9 patients exhibiting significant tumor reduction. Subsequent clinical trials indicated that [^111^In]In-labeled RPs were primarily ineffective beyond symptom alleviation, and safety apprehensions over γ-photon emission exacerbated the situation 20. The development and FDA approval of [^68^Ga]Ga-DOTATATE (NETSPOT^®^, Novartis) in 2016 and [^68^Ga]Ga-DOTATOC, which binds multiple somatostatin receptor subtypes (2, 3, and 5) with relatively higher tumor uptake, in 2019, provided improved imaging agents 196, 197. Furthermore, the approval of [^177^Lu]Lu-DOTATATE as a therapeutic partner in 2018 has largely replaced [^111^In]In-DTPA-octreotide in peptide receptor radionuclide therapy 10, 11.

#### 4.2.3 [^111^In]In-DTPA-hEGF

**Figure 6:**
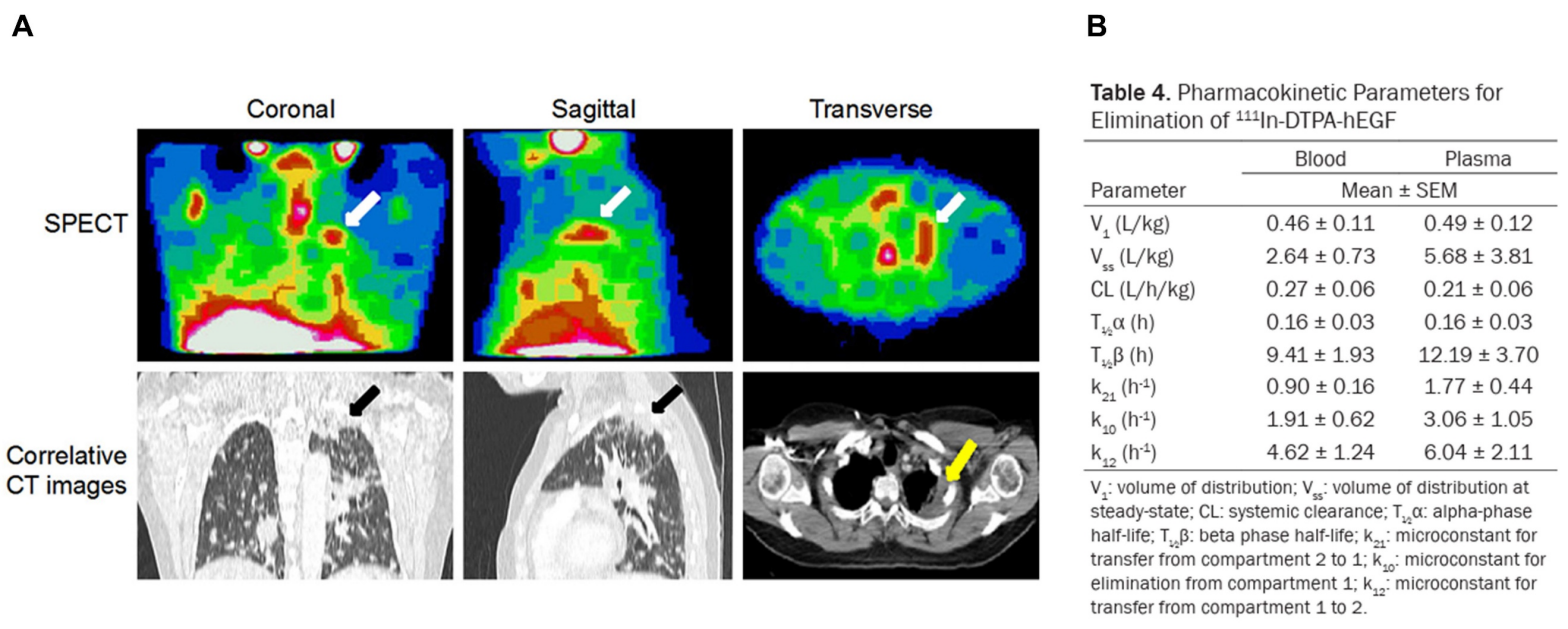
Phase I trial of [^111^In]In-DTPA-hEGF for evaluating the tumor and normal organs uptake, radiation dosimetry, and safety in patients with metastatic EGFR-positive breast cancer. (A) Representative SPECT/CT imaging from patients with metastasis to the lungs and lymph nodes showing CT images correlated with tumor accumulation of [^111^In]In-DTPA-hEGF. SPECT/CT images were acquired 24 h after injection in coronal, sagittal, and transverse planes. The tumor deposit (yellow arrow) in the left lung apex shows an accumulation of [^111^In]In-DTPA-hEGF. (B) PK parameters for the elimination of [^111^In]In-DTPA-hEGF in blood and plasma. Copyright^©^ 2014 America Journal of Nuclear Medicine and Molecular Imaging (AJMMI).

EGFR is frequently overexpressed in various epithelial-derived cancers, notably in certain breast cancer subtypes, making it an attractive therapeutic target, for which diverse small peptides and antibodies have been developed 198. Epidermal growth factor (EGF), a small peptide promoting cell growth and differentiation, binds to EGFR present on the surface of breast cancer cells and, in some cases, to rapidly proliferating cell nuclei. Reilly *et al.* produced [^111^In]-DTPA-hEGF, demonstrating its rapid binding and cellular entry in the MDA-MB-468 human breast cancer cell line expressing EGFR, with the nucleus relocating to the cell's center within 24 h. Therapeutic administered activity reaching 130 MBq resulted in cellular mortality and delivered radiation doses up to 19 Gy to the nucleus 172. Chen *et al.* administered [^111^In]In-DTPA-hEGF intravenously to MDA-MB-468 xenograft mouse models every 5 weeks, with total administered activity ranging from 27.7 to 92.5 MBq (5-17 µg), resulting in a threefold reduction in tumor growth rate compared to controls without biochemical, hematological toxicity, or body weight loss 173. Cai *et al.* discovered that DNA damage in MDA-MB-468 cells treated with [^111^In]In-DTPA-hEGF correlated with EGFR expression levels, ligand concentration, activity, and incubation duration. They proposed that γ-H2AX measurement using immunofluorescence might serve to predict and monitor therapy effects 174. Vallis *et al.* conducted the first Phase I clinical trial administering a single IV-administered activity of 370-2220 MBq (0.25 mg) of [^111^In]In-DTPA-hEGF in metastatic breast cancer patients. Whole-body radiation absorbed doses per administered activity were calculated at 0.06 mGy/MBq, corresponding to 0.133 Gy at the maximum dose. Among 15 patients, 7 (46.7%) demonstrated clear accumulation of [^111^In]In-DTPA-hEGF at breast cancer sites. No hematologic, renal, or hepatic toxicities were observed; adverse events included flushing, chills, nausea, and vomiting. One patient experienced grade 3 thrombocytopenia attributed to bone marrow metastasis rather than therapy (**Figure 6**) 22.

#### 4.2.4 [^111^In]In-DTPA-NLS-trastuzumab

**Figure 7:**
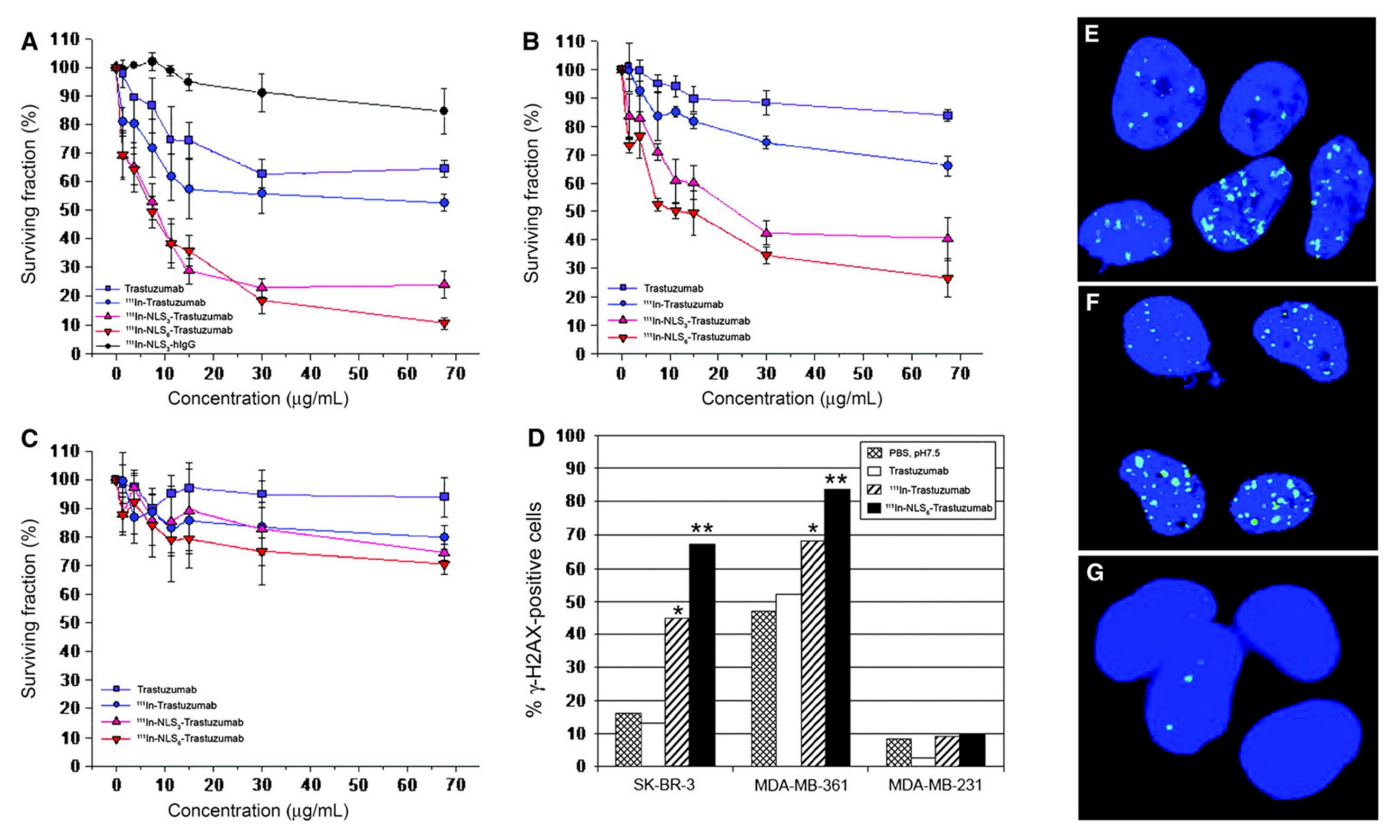
[^111^In]In-DTPA-trastuzumab with NLS as an AE-emitting RLT agent for HER2-positive breast cancer. Cell survival fraction ratios were measured in clonogenic assay for SK-BR3 (A), MDA-MB-468 (B), and MDA-MB-231 (C) cell lines treated with [^111^In]In-DTPA-NLS-trastuzumab in a dose-dependent manner. (D) Percentage of γ-H2AX positive cells reflecting DNA damage after treatment of [^111^In]In-DTPA-NLS_6_-trastuzumab. [^111^In]In-DTPA-NLS_6_-trastuzumab compared with [^111^In]In-DTPA-trastuzumab. Representative image of γ-H2AX foci (green) in SK-BR3 (E), MDA-MB-468 (F), and MDA-MB-231 (G) cell lines after treatment of [^111^In]In-DTPA-NLS_6_-trastuzumab. Nucleus stained with DAPI (blue). Copyrights^©^ 2007 Society of Nuclear Medicine and Molecular Imaging.

Radiolabeled peptides frequently induce side effects due to their inherent pharmacological features, despite the necessity for elevated dosages to provide significant therapeutic benefits. This situation has prompted the pursuit of anti-EGFR monoclonal antibodies that exhibit prolonged retention in the body and enhanced uptake efficiency. Trastuzumab (Herceptin^®^, Roche), which targets HER2, a member of the EGFR family overexpressed in 15-20% of breast tumors, is a recognized therapy 199. Constantini *et al.* developed a strategy to maximize the cytotoxic efficacy of the AE-emitting radionuclide [^111^In]In- by facilitating its proximity to DNA. A 13-amino acid NLS peptide (CGYGPKKKRKVGG) was conjugated with DTPA-trastuzumab to produce [^111^In]In-DTPA-NLS-trastuzumab. In HER2-positive SK-BR3 breast cancer cells, [^111^In]In-DTPA-NLS-trastuzumab exhibited a significantly elevated rate of receptor-mediated internalization (14.4 ± 1.8%) compared to [^111^In]In-DTPA-trastuzumab (7.2 ± 0.9%), and it was verified that it localized to the nucleus. *In vivo*, mice bearing HER2-positive MDA-MB-361 xenografts showed specific tumor nuclear uptake of 2.2-2.9 percentage of injected activity per gram (% IA/g) at 72 h post-injection of [^111^In]In-DTPA-NLS-trastuzumab, compared to 1.1% IA/g with [^111^In]In-DTPA-trastuzumab. Toxicity evaluation in Balb/c mice receiving IP-administered activity of 3.7-18.5 MBq identified a no-observed-adverse-effect level at 9.25 MBq (4 mg/kg). A single dose at this value inhibited tumor growth by approximately 4-fold relative to controls. Furthermore, two doses administered biweekly significantly improved survival compared to saline and trastuzumab controls (saline: 84 days; trastuzumab: 96 days; [^111^In]In-DTPA-NLS-trastuzumab: 140 days). Despite these promising preclinical results, clinical translation has yet to be attempted (**Figure 7**) 115.

#### 4.2.5 [^111^In]In-NLS-HuM195

**Figure 8:**
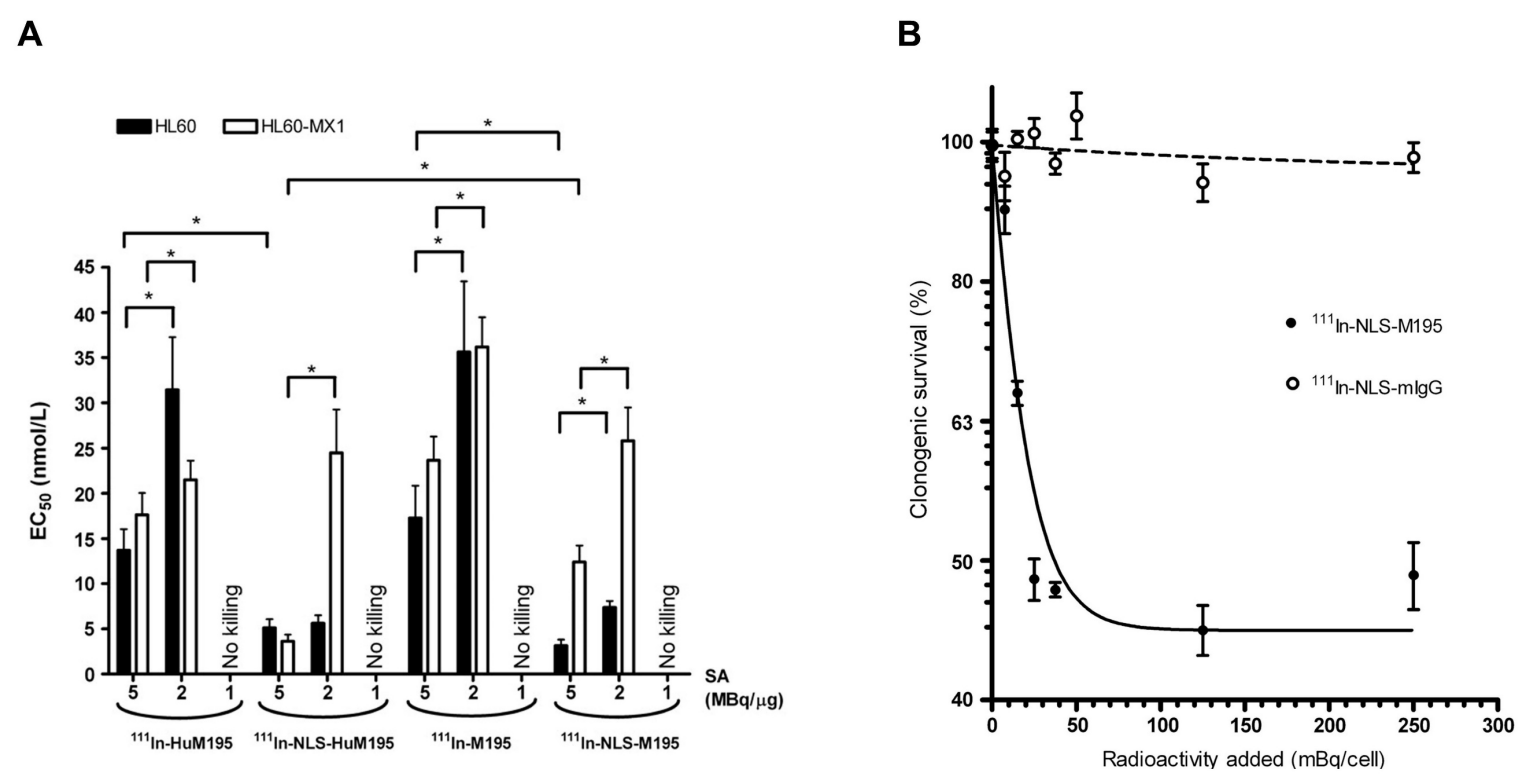
Drug-resistant AML cells and primary AML specimens are killed by [^111^In]In-NLS-HuM195. (A) Growth-inhibitory effects of HL-60 and HL-60-MX-1 cells in [^111^In]In-labeled mouse and human M195 antibody with and without NLS, at 3 different specific activity levels using the WST-1 cell viability assay. Data was expressed as mean ± S.D. of concentrations required to inhibit cell growth by 50% (EC_50_) (n = 3). (B) Percentage of clonogenic survival of HL-60-MX-1 cells after treatment of [^111^In]In-NLS-M195 or [^111^In]In-NLS-IgG with 8 MBq/µg. The data was expressed as mean ± S.D. of cell survival rates to untreated cells (n = 3). Copyright^©^ 2008 Society of Nuclear Medicine and Molecular Imaging.

CD33 is a protein expressed on approximately 85-90% of leukemic blasts, including leukemic stem cells, in AML patients, making it a promising therapeutic target 200, 201. Gemtuzumab ozogamicin (Mylotarg^®^, Pfizer Inc.) was approved by the FDA on September 1, 2017, as an antibody-drug conjugate for CD33-positive AML 202. Chen *et al.* demonstrated that targeted α-emitting RLT employing [^111^In]In-NLS-HuM195, which was engineered by modifying the anti-CD33 antibody HuM195 with simian virus 40-derived NLS peptides, successfully facilitated nuclear delivery in CD33-positive AML cells. The modified construct, [^111^In]In-NLS-HuM195, demonstrated sustained CD33 binding affinity (K_d_ = 4.3-6.9 × 10^-9^ mol/L) and a significant increase in nuclear uptake, reaching 65.9% ± 1.5% with 8 NLS peptides, compared to 10.5% ± 0.5% for the unmodified [^111^In]In-HuM195. In the HL-60 human leukemia cell line, [^111^In]In-NLS-HuM195 demonstrated significantly greater cytotoxicity compared to [^111^In]In-HuM195, with an approximate 40% reduction in the concentration required to achieve 50% inhibition of cell growth (IC_50_) (37 *vs*. 92 kBq/10^3^ cells) and 90% inhibition (IC_90_) (77-81 *vs*. 203 kBq/10^3^ cells). Cell viability assays revealed a decrease from 232 ± 22 colonies in controls to 7 ± 1 colonies at 1.48 MBq/cell, with complete colony eradication at 3.3 MBq/cell with [^111^In]In-NLS-HuM195. Importantly, no significant toxicity or organ damage was seen in mice given 3.7 MBq (22 μg) of radioconjugates 175. The preclinical results underscore the therapeutic promise of [^111^In]In-NLS-HuM195 in combating chemoresistance in AML. The results further support [^111^In]In-NLS-HuM195 as a potential AE-emitting RLT for CD33-expressing AML (**Figure 8**) 176.

### 4.3 Terbium-161

#### 4.3.1 [^161^Tb]Tb-DOTATOC

**Figure 9:**
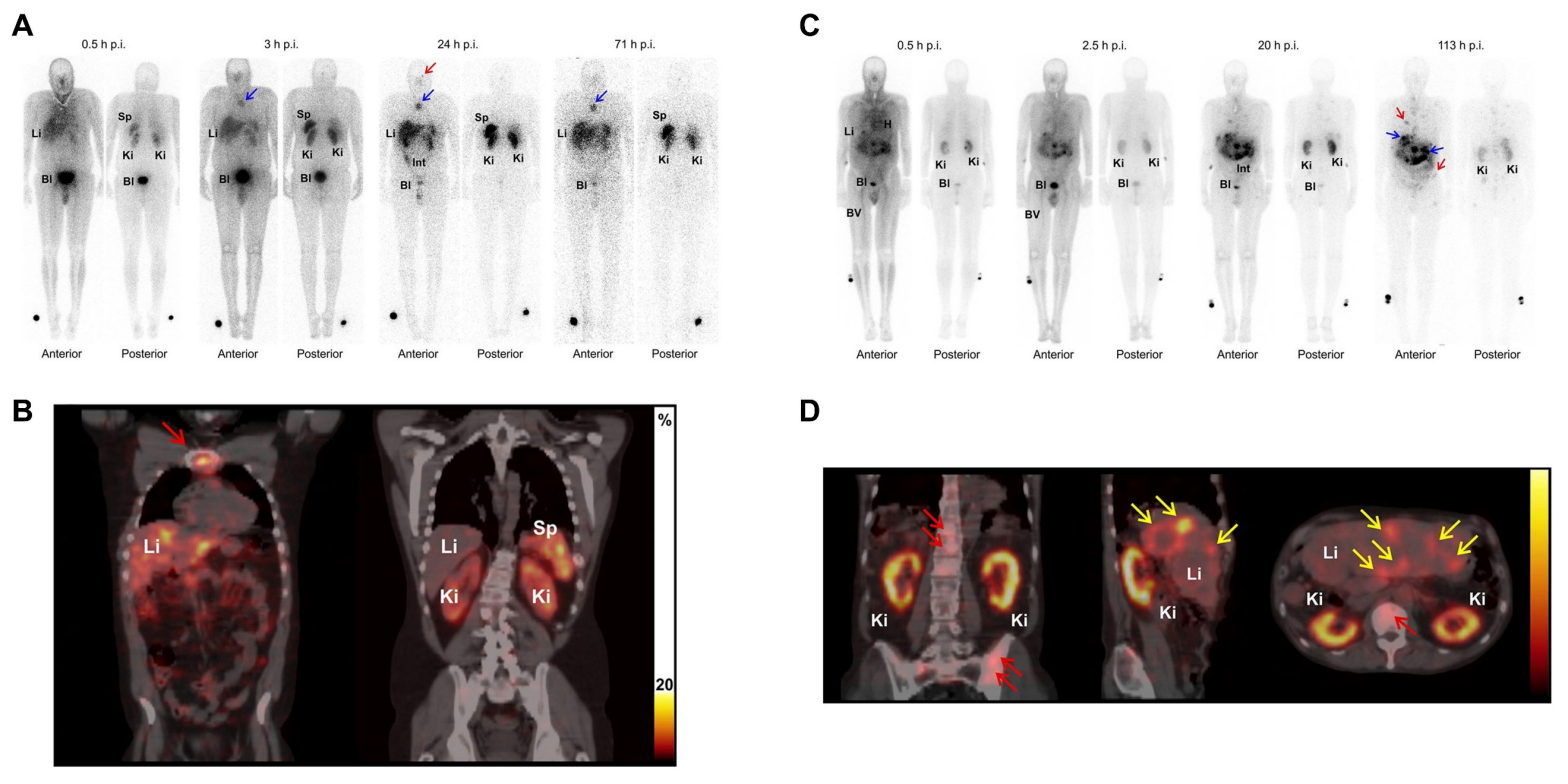
First-in-human application of [^161^Tb]Tb-DOTATOC. Whole-body imaging of patient 1 (A) and patient 2 (C) at 0.5 h, 3 h, 24 h and 71 h post-injection (p.i.). Images demonstrated physiological biodistribution of [^161^Tb]Tb-DOTATOC in liver (Li), spleen (Sp), intestines (Int), and kidneys (Ki), with excretion into urinary bladder (Bl). In patient 1, accumulations in known bone metastases (sternal manubrium [*blue arrows*] and orbital portion of the left frontal bone [*red arrows*]) were visualized. In patient 2, pathological accumulation of [^161^Tb]Tb-DOTATOC was observed in the bile ducts (*blue arrows*) and multifocal bone metastases (*red arrows*). (B) Fused coronal SPECT/CT imaging of patient 1 acquired 2 days p.i. of [^161^Tb]Tb-DOTATOC. The image shows pathologic uptake of [^161^Tb]Tb-DOTATOC in a bone metastasis (sternum [*red arrow*]). (D) Fused coronal, sagittal, and transverse SPECT/CT imaging of patient 2 at 19 h p.i. of [^161^Tb]Tb-DOTATOC. The images showed uptake of [^161^Tb]Tb-DOTATOC in the biliary tract metastases (*yellow arrows*) and in multiple osteoblastic skeletal metastases in the spine and pelvis (*red arrows*). Copyright^©^ 2021 Society Nuclear Medicine and Molecular Imaging.

Baum *et al.* conducted the first-in-human feasibility study replacing [^177^Lu]Lu-DOTATOC with [^161^Tb]Tb-DOTATOC for the treatment of neuroendocrine tumors. Two patients, a 35-year-old male with a metastatic, well-differentiated non-functioning pheochromocytoma and a 70-year-old male with a metastatic functional pancreatic neuroendocrine tumor, received 596 MBq and 1300 MBq of [^161^Tb]Tb-DOTATOC, respectively. Planar imaging and dosimetry showed time-dependent biodistribution consistent with expected accumulation in the liver, kidneys, spleen, and bladder, with visualization of small bone and liver metastases up to 71 h post-injection (**Figure 9**) 177. However, in patient 2, no splenic activity could be evaluated despite the peptide's known propensity for splenic uptake, as the patient had previously undergone splenectomy. In addition, the presence of multiple heterogeneously distributed hepatic metastases precluded reliable delineation, limiting our ability to derive quantitative liver dosimetry. Nevertheless, this study demonstrates the first-in-human feasibility of imaging even small metastatic lesions using the low activities of [^161^Tb]Tb-DOTATOC with γ-scintigraphy and SPECT/CT. Moreover, [^161^Tb]Tb-DOTATOC exhibited a distribution profile comparable to that expected for [^177^Lu]Lu-DOTATOC, and no signs of adverse effects were observed pre- to post-administration, supporting the potential of [^161^Tb]Tb-DOTATOC for theranostics use.

#### 4.3.2 [^161^Tb]Tb-DOTATATE

**Figure 10:**
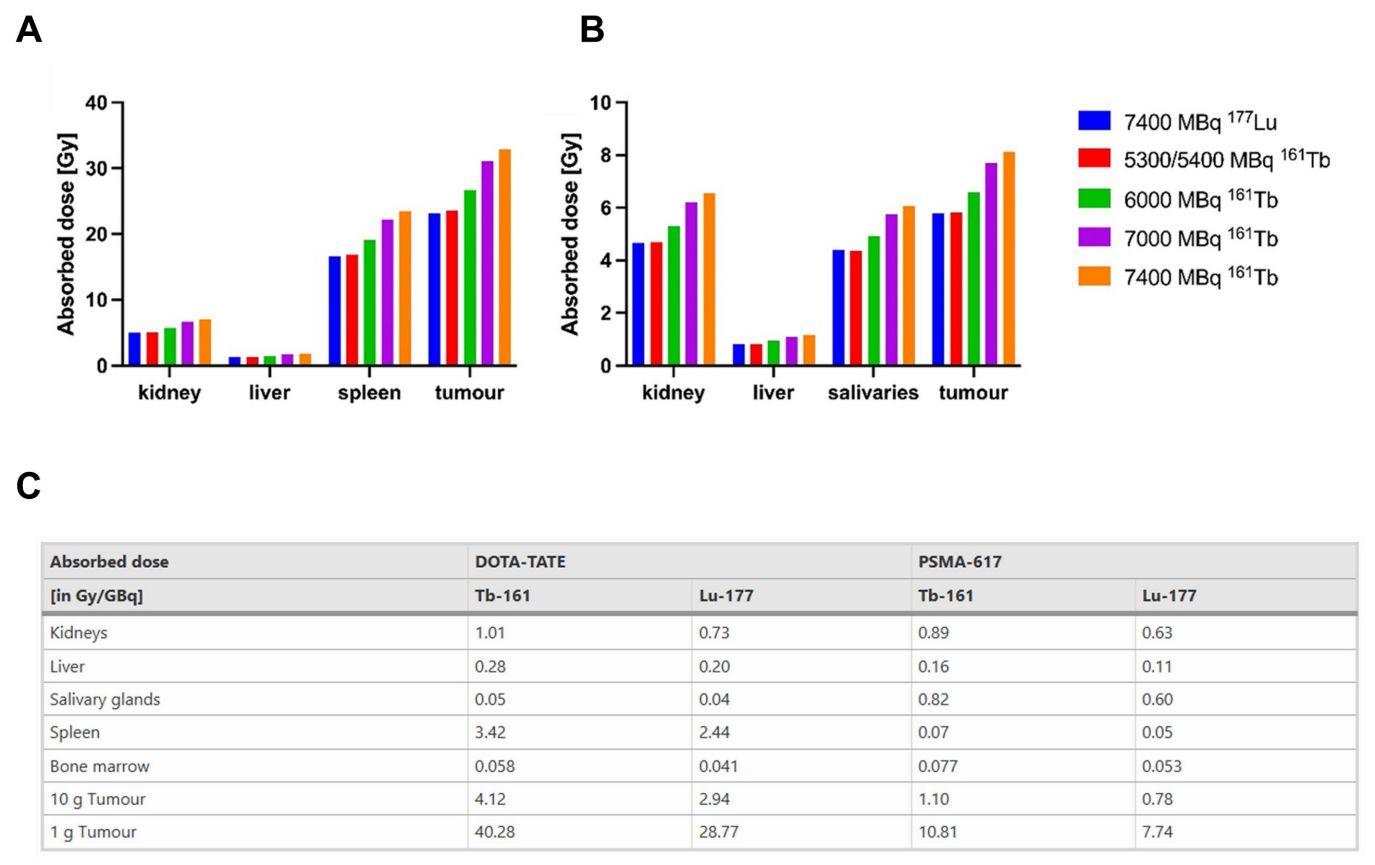
Exploring the dosimetric effect of substituting [^177^Lu]Lu- with [^161^Tb]Tb- in targeted radionuclide therapy (TRT) using the registered tracers DOTATATE and PSMA-617. (A) Absorbed dose to 10 g tumor and organs with physiological uptake by DOTATATE and (B) PSMA-617 at 7400 MBq [^177^Lu]Lu-DOTATE and various [^161^Tb]Tb- activities. (C) Absorbed doses per administered activity for DOTATATE and PSMA-617, labelled to [^161^Tb]Tb- or to [^177^Lu]Lu-, expressed as absorbed dose per administered activity (Gy/GBq). Copyright^©^ 2023 Springer Nature.

Verburg *et al.* investigated the dosimetric implications of substituting [^177^Lu]Lu- with [^161^Tb]Tb- in DOTATATE and PSMA-617-labeled RPs, employing previously defined kinetic parameters and dose assessments. Replacing [^177^Lu]Lu- with [^161^Tb]Tb- in DOTATATE resulted in a 40% increase in the absorbed dosage per administered activity to tumor tissue (10 g tumor: 2.9 Gy/GBq for [^177^Lu]Lu-DOTATATE against 4.1 Gy/GBq for [^161^Tb]Tb-DOTATATE). However, the dose to dose-limiting organs, such as the kidneys and bone marrow, also increased by 39% (0.73 Gy/GBq *vs*. 1.01 Gy/GBq) and 42% (0.04 Gy/GBq *vs*. 0.06 Gy/GBq), respectively. To maintain equivalent non-target organ doses and avoid increased toxicity, a standard administered activity of 7.4 GBq [^177^Lu]Lu-DOTATATE per treatment cycle should be reduced to approximately 5.4 GBq [^161^Tb]Tb-DOTATATE. At these adjusted activities, the absorbed dose to a 10 g tumor remains comparable or slightly higher (22.3 Gy *vs*. 21.8 Gy). Similarly, for PSMA-TRT, [^161^Tb]Tb-DOTATATE increased tumor absorbed dose by 40% (0.8 Gy/GBq *vs*. 1.1 Gy/GBq), with concomitant increases in kidney (38%) and bone marrow (46%) doses. Thus, to achieve equivalent normal organ damage, 7.4 GBq of [^177^Lu]Lu-DOTATATE TRT should be replaced with about 5.3 GBq of [^161^Tb]Tb-DOTATATE, therefore enhancing tumor dose delivery without increasing normal tissue toxicity (**Figure 10**) 178. A lower standard administered activity is also advantageous from a radiochemical standpoint, as reduced total activity generally improves radiochemical stability. From a radiation safety perspective, [^161^Tb]Tb-, whose γ-emission spectrum peaks at 43.1 keV and 74.6 keV, is markedly more favorable than [^177^Lu]Lu-, which exhibits higher-energy peaks at 112.9 keV and 208.4 keV. Moreover, the lower total activity required to achieve an equivalent absorbed dose to the target increases the feasibility of performing such treatment in an outpatient procedure.

#### 4.3.3 [^161^Tb]Tb-DOTA-cm09

**Figure 11:**
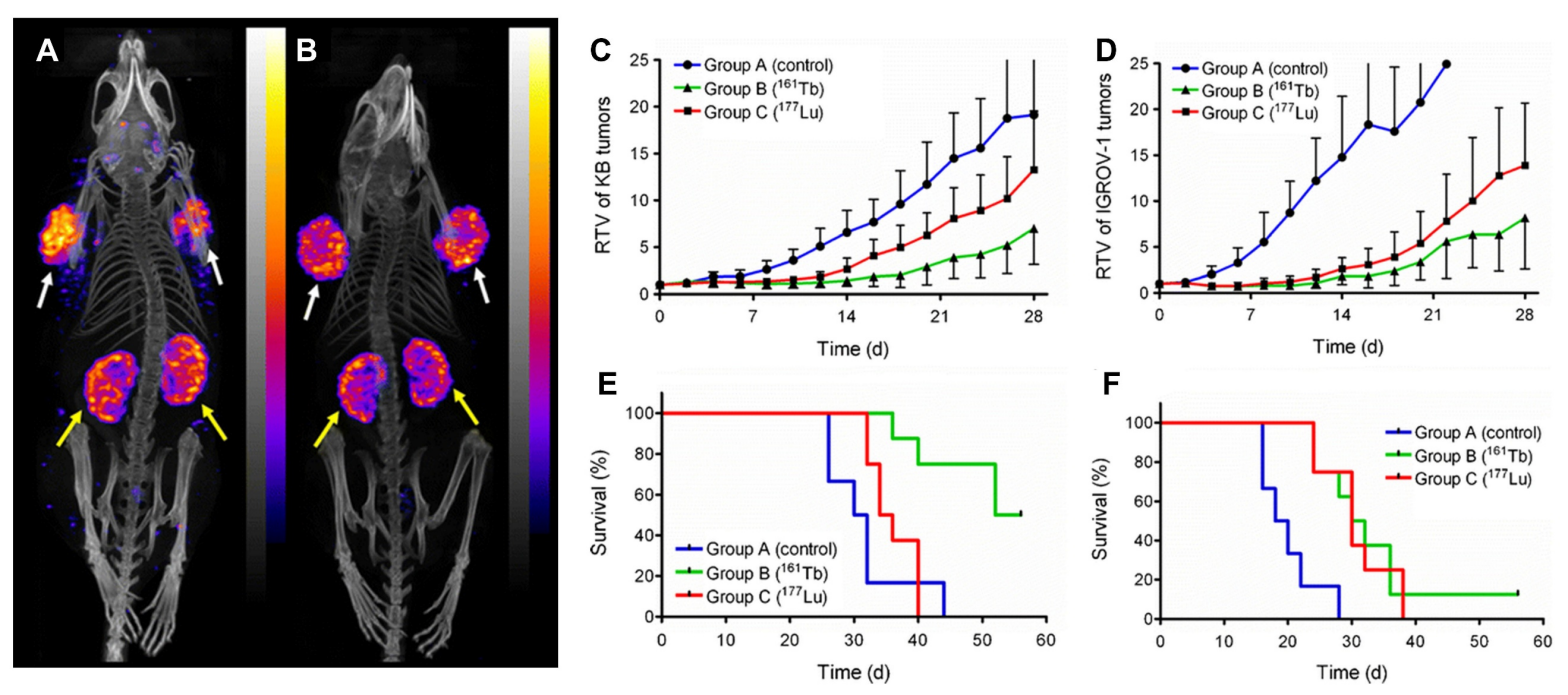
Direct *in vitro* and *in vivo* comparison of [^161^Tb]Tb- and [^177^Lu]Lu-labeled using a tumor-targeting folate conjugate (cm09). Representative *in vivo* SPECT/CT images of KB tumor-bearing mice following administration of [^161^Tb]Tb-DOTA-cm09 (A) and [^177^Lu]Lu-DOTA-cm09 (B). The tumor and kidneys are indicated with *white* and *yellow arrows*. Relative tumor volumes (RTV) of KB tumor-bearing mice (C) and IGROV-1 tumor-bearing mice (D). Percentage of survival of KB tumor-bearing mice (E) and IGROV-1 tumor-bearing mice (F). Copyright^©^ 2014 Springer Nature.

Müller *et al.* developed a DOTA-folate conjugate (cm09) targeting the folate receptor (FR), which is overexpressed in certain tumors, and compared [^177^Lu]Lu-DOTA-cm09 and [^161^Tb]Tb-DOTA-cm09 *in vitro* and *in vivo*. To achieve maximal inhibition of tumor cell survival half-life, KB cells required approximately 4.5-fold lower radioactivity concentration with [^161^Tb]Tb-DOTA-cm09 compared to [^177^Lu]Lu-DOTA-cm09 (IC_50_ ~0.014 MBq/mL *vs*. ~0.063 MBq/mL), while IGROV-1 cells showed a 1.7-fold difference (IC_50_ ~2.53 MBq/mL *vs*. ~4.52 MBq/mL). SPECT imaging demonstrated similar image quality between the two radioligands. In murine models with tumors, [^161^Tb]Tb-DOTA-cm09 showed superior efficacy against tumors compared to [^177^Lu]Lu-DOTA-cm09, irrespective of tumor type. In KB tumor-bearing mice, the mean survival durations were 31 days for controls, 35 days for [^177^Lu]Lu-DOTA-cm09, and 54 days for [^161^Tb]Tb-DOTA-cm09. In IGROV-1 tumor-bearing mice, life was extended to 30 and 31 days for [^177^Lu]Lu-DOTA-cm09 and [^161^Tb]Tb-DOTA-cm09, respectively, compared to 19 days in the control group. No hematologic, hepatic, or renal toxicities were observed following treatment (**Figure 11**) 179.

#### 4.3.4 [^161^Tb]Tb-SibuDAB

Tschan *et al.* developed the (*S*)-isomer of [^177^Lu]Lu-Ibu-DAB-PSMA, termed [^177^Lu]Lu-SibuDAB, and its labeled counterpart, [^161^Tb]Tb-SibuDAB, featuring albumin-binding properties to enhance blood circulation and tumor uptake relative to conventional PSMA-targeted radioligands 180. Biodistribution studies showed that blood retention of [^161^Tb]Tb-SibuDAB at 4 h post-injection was significantly higher (6.5 ± 3.7% IA/g) than that of [^161^Tb]Tb-PSMA-I&T (0.02 ± 0.01% IA/g). Consequently, tumor uptake of [^161^Tb]Tb-SibuDAB (75 ± 5% IA/g) was nearly twice that of [^161^Tb]Tb-PSMA-I&T (42 ± 14% IA/g). In preclinical tumor models, all [^161^Tb]Tb-labeled PSMA ligands significantly delayed tumor growth compared to their [^177^Lu]Lu-labeled counterparts. Treatment of mice with 10 MBq of [^161^Tb]Tb-SibuDAB resulted in complete tumor regression over two observation periods, whereas one mouse treated with 10 MBq of [^177^Lu]Lu-SibuDAB showed tumor regrowth approximately six weeks post-treatment. Body weights of treated mice remained comparable to age-matched untreated controls at euthanasia, and histopathological analyses revealed no hematologic or organ toxicities 181.

#### 4.3.5 [^161^Tb]Tb-PSMA-617

**Figure 12:**
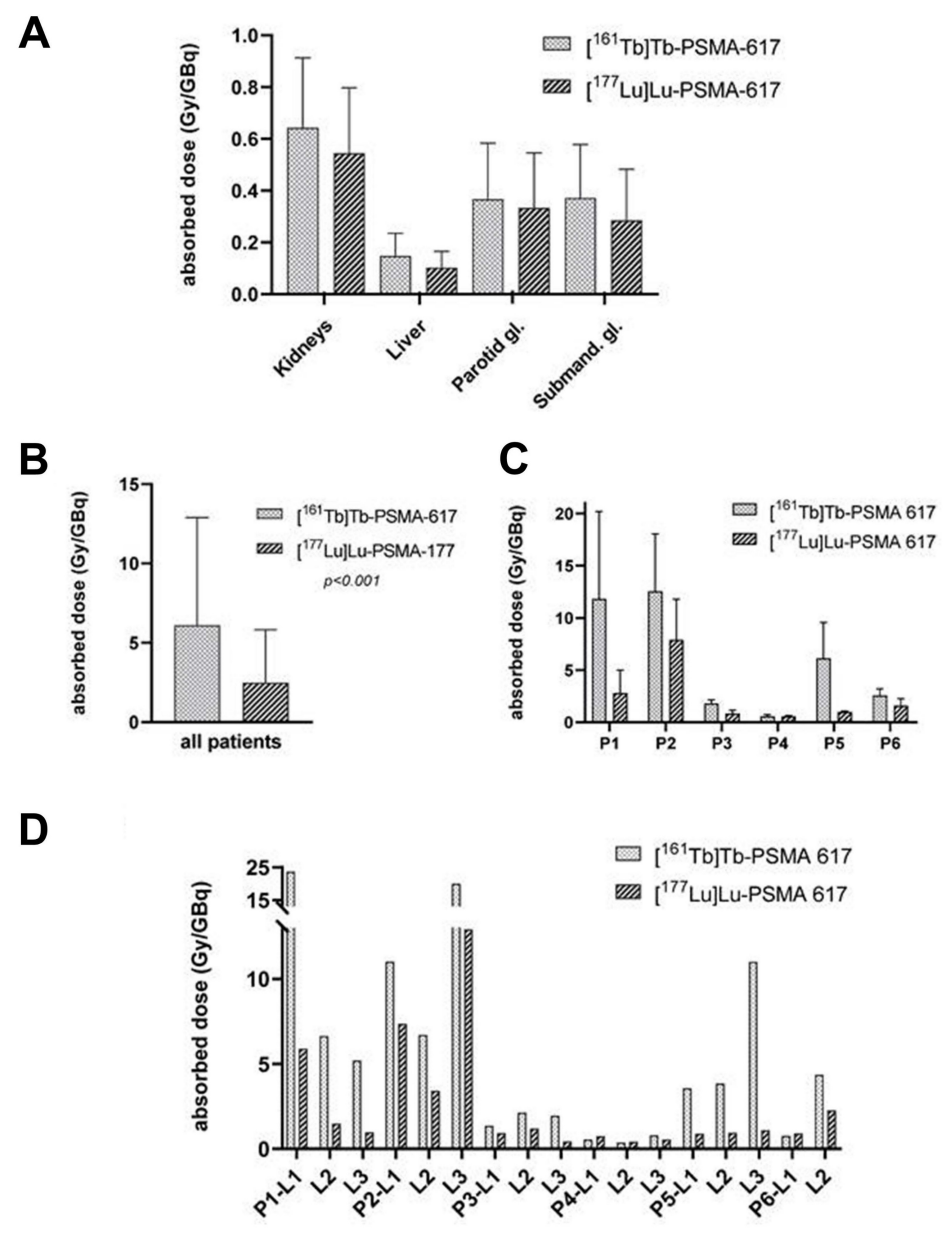
Head-to-head comparison between [^161^Tb]Tb-PSMA-617 and [^177^Lu]Lu-PSMA-617, including preclinical dosimetry results in patients with mCRPC. (A) Mean absorbed doses (Gy/GBq) accumulated to kidneys, liver, parotid glands and submandibular glands determined for [^161^Tb]Tb-PSMA-617 and [^177^Lu]Lu-PSMA-617 over all patients. (B) Absorbed doses in tumor lesions determined after application of [^161^Tb]Tb-PSMA-617 and [^177^Lu]Lu-PSMA-617, respectively. (C) Mean tumor lesion absorbed dose per patient, and (D) absorbed dose in each individual tumor lesion. Copyrights^©^ 2024 IVYSPRING.

[^177^Lu]Lu-PSMA-617 (Pluvicto^®^, Novartis) has received FDA approval for the treatment of mCRPC, and its [^161^Tb]Tb- analog, [^161^Tb]Tb-PSMA-617, is currently being evaluated in the ongoing Phase I REALITY trial (NCT04833517). Schaefer-Schuler *et al.* reported on six mCRPC patients who prospectively received [^161^Tb]Tb-PSMA-617 at a mean activity of 6.4 ± 1.2 GBq. The mean absorbed dose per GBq in tumor lesions was significantly higher with [^161^Tb]Tb-PSMA-617 (6.10 ± 6.59 Gy/GBq) than with [^177^Lu]Lu-PSMA-617 (2.59 ± 3.30 Gy/GBq), and tumor effective half-lives were also longer (46.1 ± 19.2 h *vs*. 35.3 ± 6.3 h). Among 17 evaluated lesions, 14 (82.4%) absorbed more radiation from [^161^Tb]Tb-PSMA-617 than for [^177^Lu]Lu-PSMA-617. Despite higher tumor doses, normal organ radiation exposure remained comparable between both tracers. The mean therapeutic index for kidneys markedly increased with [^161^Tb]Tb-PSMA-617 (11.54 ± 9.74) compared to [^177^Lu]Lu-PSMA-617 (5.28 ± 5.13), suggesting a more advantageous therapeutic window (**Figure 12**) 182. Following one cycle, three patients saw a decrease in PSA levels by 18.6%, reaching 53.4%, whilst the other three exhibited an increase of 18.0%, culminating at 73.2%.

#### 4.3.6 [^161^Tb]Tb-DOTA-LM3

**Figure 13:**
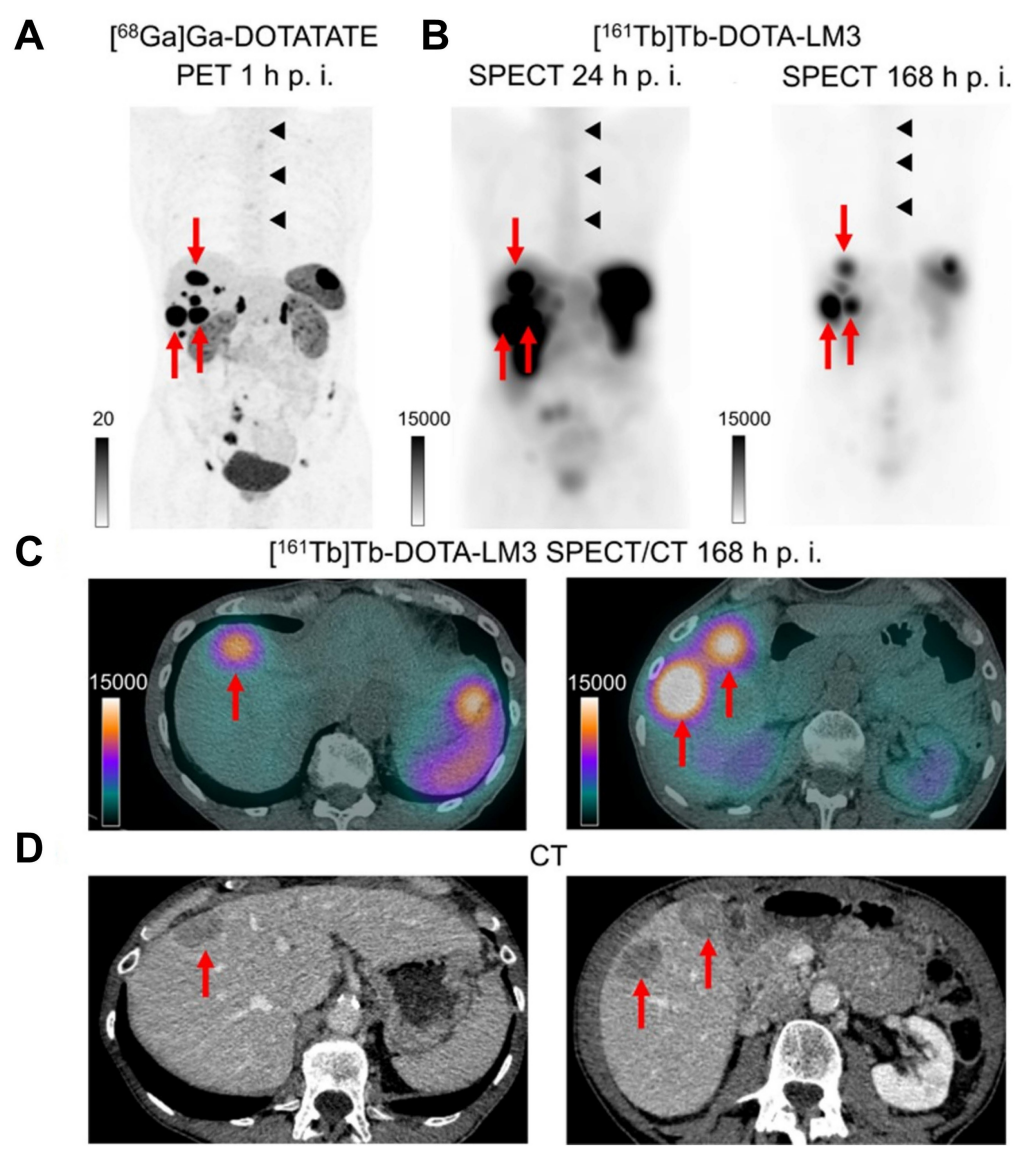
First-in-human administration of [^161^Tb]Tb-labeled SSTR-2 antagonist ([^161^Tb]Tb-DOTA-LM3) in a patient with a metastatic NET of the ileum. (A) Representative PET imaging after 1 h post-administration of [^68^Ga]Ga-DOTATATE. (B) Representative SPECT imaging after 24 and 168 h post-administration of [^161^Tb]Tb-DOTA-LM3. (C) Transverse SPECT image 168 h post-injection of [^161^Tb]Tb-DOTA-LM3 and correlated CT image (D). The accumulated tumor area signal was indicated by the *red arrow*. Copyrights© 2024 Springer Nature.

In a cohort of 51 neuroendocrine tumor patients, [^177^Lu]Lu-DOTA-LM3, an SSTR2-targeting antagonist, exhibited superior tumor uptake compared to [^177^Lu]Lu-DOTATOC, without causing significant acute toxicity 204. Based on this, Borgna *et al.* compared the therapeutic efficacy and dosimetry of [^161^Tb]Tb- *vs*. [^177^Lu]Lu-labeled DOTA-LM3 and DOTATOC in SSTR-positive AR42J pancreatic tumor models. Notably, [^161^Tb]Tb-DOTA-LM3 exhibited a remarkable 102-fold increase in cytotoxicity relative to [^177^Lu]Lu-DOTA-LM3 (EC_50_ = 0.001 MBq/mL *vs*. 0.102 MBq/mL) and was about 820 times more potent than the clinical agent [^177^Lu]Lu-DOTATOC. [^161^Tb]Tb-DOTA-LM3 induced a greater incidence of DNA DSBs (about 8% γ-H2AX+ cells compared to around 3%) and significantly elevated tumor-to-kidney ratios. Biodistribution investigations revealed sustained and elevated tumor uptake at 24 h (35 ± 7% IA/g) and 48 h (21 ± 4% IA/g), exceeding [^161^Tb]Tb-DOTATOC at all evaluated time periods. *In vivo*, [^161^Tb]Tb-DOTA-LM3 significantly inhibited tumor growth (44 ± 5 days) compared to [^177^Lu]Lu-DOTA-LM3 (35 ± 7 days), with no signs of liver toxicity 183. The first-in-human application in a patient with a metastatic ileal NET confirmed high-quality SPECT/CT up to 168 h post-injection and favorable dosimetry, including a tumor absorbed dose of 28 Gy/GBq, supporting its further evaluation in a dose-escalation Phase 0B trial (NCT05359146) (**Figure 13**) 184.

The summarized studies on the prior development of AE-emitting RLTs demonstrate substantial potential and insights; however, when compared with the existing limitations and potential mitigation strategies detailed in **Section 3**, several notable common implications emerge. Many early AE clinical trials failed to provide enduring therapeutic effects, despite promising results in animal studies. This phenomenon illustrates the significance of precision in targeting cells, maneuvering them within cells, and directing them to the nucleus or perinuclear region. Furthermore, considerations such as accelerated systemic clearance and detrimental PK characteristics often limited therapeutic outcomes. Comparing α- and β-emitting RLTs reveals that certain issues in the translational process are prevalent across all radionuclide series. Nonetheless, the limited range of AE emissions and the consequent severe spatial constraints on cytotoxicity may render these issues more apparent. A thorough evaluation of unintended off-target toxicity in normal tissues resulting from insufficient nuclear delivery will be essential for the safe clinical implementation of AE-emitting RLTs. Contextualizing these discoveries within the broader RLT ecosystem reveals both opportunities and obstacles. Successful AE translation necessitates advancements in vector engineering, refined radiochemical techniques to enhance PK, dosimetry, and accurate intracellular localization. The integration of these multidisciplinary approaches can facilitate the intelligent advancement of next-generation AE-emitting RLTs with enhanced therapeutic efficacy.

## 5. Imaging Distribution of AE/Companion Emissions and Suitable/Potential Pairs for Diagnostics Imaging

### 5.1 Gallium-67 / -68

Gallium-67 ([^67^Ga]Ga-), with a physical half-life of 78.2 h, decays exclusively through EC, releasing an average of 4.9 AEs per decay with a mean energy width of 6.3-6.6 keV, as well as up to ten γ-photons. The major γ-emissions, 93 keV (39%), 185 keV (21%), and 300 keV (15%), are well suited for SPECT imaging. In comparison, Gallium-68 ([^68^Ga]Ga-), which has a much shorter half-life of 67.7 min, emits positrons with an average energy of 830 keV and is a widely utilized PET radiotracer. [^67^Ga]Ga has been utilized in diagnostic imaging for an extended period; nonetheless, there has been significant recent interest in its potential application as a therapeutic radionuclide, particularly due to its emission of AEs 57. Compared to [^111^In]In-, which has a similar half-life (67.2 h) and AE energy (6.8 keV), [^67^Ga]Ga- emits approximately one-third the number of AEs per decay (4.9 *vs*. 14.7) yet still offers promising therapeutic applications when high subcellular localization is achieved. Furthermore, [^67^Ga]Ga- and [^68^Ga]Ga- represent a promising theranostic pair when labeled with the same targeting vector, such as [DFO]-Octreotide, enabling multimodal imaging, SPECT with [^67^Ga]Ga- and PET with [^68^Ga]Ga-, for the management of somatostatin receptor-positive neuroendocrine tumors 204. This dual capability highlights the expanding role of gallium isotopes in precision nuclear medicine.

### 5.2 Copper-64 / -67

Copper-64 ([^64^Cu]Cu-) possesses a half-life of 12.7 h and exhibits diverse degradation trends. For PET imaging, it emits positrons (17.6%); for AEs generation, it emits EC (43.9%); and for β⁻ emission, it emits β⁻ (38.5%). The relatively low positron energy (about 278 keV), almost one-third that of [^68^Ga]Ga-, facilitates higher resolution in PET imaging. Combined with its longer circulation time, [^64^Cu]Cu- is particularly well suited for immuno-PET applications targeting slower-accumulating biological vectors such as monoclonal antibodies. Copper-67 ([^67^Cu]Cu-), on the other hand, is a therapeutic radionuclide with a longer half-life (61.9 h) and emits β⁻ (mean: 141 keV; max: 562 keV) particles with an energy profile similar to that of [^131^I]-, along with γ-emissions at 93 keV and 185 keV suitable for SPECT-based imaging and dosimetry. As a theranostics pair, [^64^Cu]Cu- and [^67^Cu]Cu- have demonstrated promising results. In clinical studies involving 36 patients with suspected metastatic or primary colorectal cancer, the [^64^Cu]Cu-BAT-2IT-1A3 monoclonal antibody exhibited more tumor-specific uptake than [^18^F]FDG 205. Preclinical experiments involving GW39 human colorectal cancer xenografts in hamsters have shown that both [^64^Cu]Cu- and [^67^Cu]Cu-labeled BAT-2IT-1A3 markedly reduced tumor burden without inducing systemic toxicity. This study validated that the copper-based RP platform is applicable for both diagnosis and treatment 206.

### 5.3 Mercury-197 / Mercury-197m

The ground and metastable isotopes of mercury, [^197^Hg]Hg- (half-life: 64.1 h) and [^197m^Hg]Hg- (t_1/2_ = 23.8 h), are attracting renewed interest as promising theranostic radionuclides. [^197^Hg]Hg- emits an average of 23.2 AEs per decay with a total mean energy of 7.6 keV, whereas [^197m^Hg]Hg- releases 19.4 AEs at 7.4 keV via internal transition and EC processes. Their respective γ-emissions, particularly at 77.35 keV and 133.98 keV, support the feasibility of SPECT imaging in addition to therapeutic applications 207. These isotopes provide a unique opportunity to combine theranostics functionality within a single radionuclide, enabling precise monitoring and personalized treatment adaptations. Nonetheless, the neurotoxicity of mercury, its bioaccumulation in tissues, and the limited accessibility of chelation therapy have complicated its clinical use 208. Recent advancements in cyclotron-based synthesis techniques, particularly the ^197^Au(p,n)^197m^Hg reaction, have enabled the production of [^197^Hg]Hg- with a high molar activity (~500 GBq/µmol). This significantly mitigates the dangers associated with elemental mercury 209. Concurrently, research into biologically safe and compatible chelators is enabling targeted vectorization, and although the clinical use of mercury radionuclides was once abandoned, the refined understanding of their decay properties and imaging potential now positions [^197^Hg]Hg-/[^197m^Hg]Hg- as viable agents for future theranostic applications in cancer imaging.

### 5.4 Cerium-134/Lanthanum-134 -Actinium-225

The [^134^Ce]Ce/[^134^La]La- theranostic pair has attracted significant interest as a chemically congruent surrogate for [^225^Ac]Ac-based targeted alpha therapy. [^134^Ce]Ce- (t_1/2_ = 3.2 days) undergoes EC decay to form [^134^La]La- (t_1/2_ = 6.5 min), which emits high-energy positrons (69%, 2.69 MeV) suitable for PET imaging. This *in vivo* generation of a PET-emitting daughter from a longer-lived parent offers a unique strategy for real-time imaging of biological distribution and tumor targeting. In contrast to conventional diagnostic surrogates like [^68^Ga]Ga- or [^89^Zr]Zr-, which are limited by quick degradation or inconsistent chelation properties, [^134^Ce]Ce- offers a chemically and kinetically analogous alternative to [^225^Ac]Ac- 210, 211. The analogous trivalent charge state and coordination characteristics enable the utilization of identical targeting vectors and chelators (such as DTPA, DOTA, and 6-[[16-[(6-carboxypyridin-2-yl)methyl]-1,4,10,13-tetraoxa-7,16-diazacyclooctadec-7-yl]methyl]pyridine-2-carboxylic acid (MACROPA), facilitating precise predictions of PK and dosimetry 212, 213. Moreover, the low recoil energy (< 0.2 eV) associated with AE emission in [^134^Ce]Ce- decay prevents detachment of the daughter nuclide ([^134^La]La-) from the chelator complex, preserving imaging fidelity 214. These advantages position [^134^Ce]Ce-/[^134^La]La- as an ideal PET-compatible partner for therapeutic [^225^Ac]Ac-, addressing key limitations in the current theranostic paradigm.

Recent research conducted by Bobba *et al.* has elucidated the increasing importance of the [^134^Ce]Ce/[^134^La]La- pair as a diagnostic analog for [^225^Ac]Ac-based TRT. In their study, the CD46-targeted monoclonal antibody YS5 was conjugated with DOTA and MACROPA chelators and subsequently radiolabeled with [^134^Ce]Ce-, enabling a direct comparison of *in vivo* distribution to that of free ^134^CeCl₃ 213. The hepatic and osseous uptake of both [^134^Ce]DOTA and [^134^Ce]MACROPA conjugates was significantly diminished at 1 h post-injection (liver: 0.44 ± 0.13 and 0.80 ± 0.15% IA/g; bone: 0.32 ± 0.13 and 0.39 ± 0.09% IA/g, respectively) in contrast to free ^134^CeCl₃ (22.79 ± 1.75 and 13.06 ± 0.40% IA/g). These results suggest effective chelation and PK congruence with [^225^Ac]Ac-labeled constructs. The same group extended this strategy to label the small-molecule RP PSMA-617 with [^134^Ce]Ce-, and when administered to nude mice bearing PSMA-positive PC3-PIP tumors, [^134^Ce]Ce-PSMA-617 exhibited high tumor uptake (5.64 ± 1.86% IA/g at 4 h) with slow clearance over 72 h 215. Beyond imaging, the therapeutic potential of [^134^Ce]Ce-PSMA-617 was evaluated in tumor-bearing mice administered with 37 or 111 MBq, both doses producing marked tumor growth inhibition and extended survival compared with control animals. Notably, no histopathological changes were detected in major organs, demonstrating the compound's safety at diagnostic activity levels. These results support the use of [^134^Ce]Ce-/[^134^La]La- as a PET-based surrogate for preclinical assessment of [^225^Ac]Ac-labeled therapeutics.

### 5.5 Terbium-152, 155 / Terbium-161

Terbium is a promising member of the lanthanide series in nuclear medicine, gaining attention as a versatile theranostic radionuclide family, much like the FDA-approved lutetium isotopes. This flexible theranostic radionuclide family is garnering significant attention, similar to the FDA-approved lutetium isotopes. Terbium possesses four isotopes of medical significance: [^149^Tb]Tb-, [^152^Tb]Tb-, [^155^Tb]Tb-, and [^161^Tb]Tb-. All these isotopes exhibit identical chemical characteristics, rendering terbium an excellent choice for the development of matched diagnostic and therapeutic pairs 216, 217. [^161^Tb]Tb- (t_1/2_ = 6.89 d) is a promising radionuclide for theranostics applications because it emits β- particles (E_β-_ = 154 keV) with a mean energy comparable to that of [^177^Lu]Lu- (t_1/2_ = 6.65 d, E_β-_ = 134 keV), while also releasing substantial AE and CE that enhance localized cytotoxicity 216. In particular, [^161^Tb]Tb- delivers AE with a total energy of 5.1 keV and an average energy of 5.7 keV per decay, providing enhanced subcellular radiotoxicity 218. [^161^Tb]Tb- has superior IC and AE emission energies compared to [^177^Lu]Lu-, with mean energies of 39.28 keV for E_ICmean_ and 8.94 keV for E_AEmean_ in [^161^Tb]Tb, *vs*. 13.52 keV for E_ICmean_ and 1.13 keV for E_AEmean_ in [^177^Lu]Lu-. This indicates that it may increase cellular toxicity 219, 220. Due to these attributes, [^161^Tb]Tb- is garnering significant interest as a potential substitute for [^177^Lu]Lu-, a prevalent β-emitting radionuclide, in precision radiotherapy, particularly for small lesions or micrometastases 86, 221. The radionuclide also emits γ-rays at energies of approximately 45, 49, and 75 keV, making it suitable for SPECT imaging 222. However, although [^161^Tb]Tb- in principle emits multiple low-energy γ-photons that enable SPECT, the resultant image quality is inherently limited by the low photon abundance and suboptimal imaging energies. These constraints diminish quantitative accuracy and hinder the high-resolution visualization necessary for therapy planning. Therefore, use in conjunction with a dedicated diagnostic terbium isotope provides a more reliable theranostic framework for precise dosimetry and treatment optimization.

[^149^Tb]Tb- (t_1/2_ = 4.12 h) is the only α-emitting terbium (16.7%, 3.967 MeV) isotope, emitting α-particles with a short range (25-28 μm) and high LET (~140-142 keV/μm), and is therefore ideal for targeted alpha therapy 223. However, its fundamentally different decay physics compared with [^161^Tb]Tb- (i.e., different emitted particle types), together with its relatively low positron branching ratio, still yields limited PET image quality 223, 224. [^152^Tb]Tb- (t_1/2_ = 17.5 h) decays to [^152^Gd]Gd- by β^+^ decay (E_β+_ = 1.14 MeV, 20.3%) and EC (79.3%), and thus can serve as a PET companion isotope for [^161^Tb]Tb-. To characterize *in vivo* lanthanide kinetics, Beyer *et al.* showed that [^152^Tb]Tb- can yield superior PET image quality compared to [^149^Tb]Tb- 225. Nevertheless, the high positron energy and low intensity may negatively affect image quality. In addition, the simultaneous emission of multiple γ-rays complicates imaging: while this makes [^152^Tb]Tb- a potential candidate for SPECT as well, it also imposes limitations on its use as a PET isotope. Although proton-induced spallation enables the repeated production of no-carrier-added [^152^Tb]Tb-, suitable for preclinical studies, current production capacities remain insufficient to supply the large quantities required for clinical translation 226. Consequently, [^155^Tb]Tb, whose production routes are more favorable for clinical application and will be discussed below, has emerged as a promising diagnostic terbium radioisotope.

[^155^Tb]Tb- (t_1/2_ = 5.23 d) proceeds to decay exclusively via EC to [^155^Gd]Gd- and emits low-energy γ-rays (E_γ_ = 86.6 keV, 32.0%; 105.3 keV, 25.1%) and X-rays (E_X_ = 45 keV [± 11%], 107 keV). This renders it ideal for SPECT imaging. The extended half-life reflects the PK characteristics of [^161^Tb]Tb-, facilitating thorough *in vivo* biodistribution studies. Wharton *et al.* evaluated [^155^Tb]Tb- and [^161^Tb]Tb-radiolabeled Crown-TATE in SSTR2-positive AR42J tumor-bearing mice, showing efficient radiolabeling, high serum stability (> 99.5% radiochemical purity over 7 days), and SSTR2-specific targeting. Longitudinal SPECT/CT revealed tumor uptake of 32.6% IA/g for [^155^Tb]Tb-Crown-TATE and 30.0% IA/g for [^161^Tb]Tb-Crown-TATE at 2.5 h post injection, corroborated by biodistribution studies 227. Also, they reported the first preclinical evaluation of a matched terbium theranostics pair for Crown-melanoma using alpha-melanocyte stimulating hormone radiolabeled with [^155^Tb]Tb- for SPECT imaging and [^161^Tb]Tb- for TRT. Both tracers exhibited similar tumor uptake (~6-7% IA/g) in melanocortin-1 receptor-positive B16-F10 melanoma-bearing mice, with minimal off-target retention 228. Koniar *et al.* demonstrated that preclinical SPECT imaging of both [^155^Tb]Tb- and [^161^Tb]Tb- is feasible with high spatial resolution (< 0.85 nm) using a high-resolution collimator, as shown in phantom studies. Importantly, their quantitative assessments, measuring recovery coefficients and contrast-to-noise ratio, supported the use of this imaging approach to inform PK and dosimetry in matched theranostics development 229.

## 6. Prospects for the future

Future progress in AE-emitting RLTs will stem from the amalgamation of achievements across multiple disciplines rather than from singular technological innovations. This encompasses bioorthogonal conjugation with click chemistry to assemble radionuclide payloads and targeting vectors *in vivo*, delivery systems that efficiently transport radioisotopes to the nucleus or cellular organelles, computational tools that precisely forecast DNA proximity binding, and mechanism-driven combination therapies that effectively eradicate tumor cells while minimizing genotoxicity to normal tissues. Each area of advancement is promising individually, but the ultimate therapeutic outcome will rely on the effective integration of chemistry, delivery mechanisms, radiophysical dosimetry, and biological understanding.

Bioorthogonal click chemistry and pretargeting techniques offer a feasible approach for therapeutic AE-emitting RLTs by distinguishing the biological targeting phase from the radioactivity administration phase. This is particularly advantageous for short-range, highly localized emitters with restricted therapeutic windows. Rapid IEDDA pairings, such as tetrazine-TCO, SPAAC variations, and other catalyst-free ligations, have improved significantly in radiochemistry. They exhibit superior *in vivo* reaction kinetics and less off-target accumulation in pretargeted theranostic models. To effectively modify these tactics for AE-emitting radionuclides, it is crucial to improve reactivity, steric considerations, and PK 146.

Combination therapies employing DNA-damage mechanisms are poised to provide the first substantial clinical applications of AE-emitting RLTs. PARP-targeted small compounds or ligands that release AE-emitting radionuclides and bind to PARP have demonstrated significant chromatin-proximal DNA damage and effective tumor suppression in preclinical settings. This method is intriguing given PARP inhibitors are already utilized in clinical settings, and the scaffold's chemistry is manageable. Concurrently, substantial research on radiosensitizers, including comprehensive preclinical and early clinical studies including PARP inhibitors, provides a rational foundation for sequencing and dosing. The heightened danger of off-target genomic damage necessitates a comprehensive evaluation of normal-tissue genotoxicity in combination trials 110, 230.

Beyond PARP, combinatorial strategies with classical radiosensitizers, replication-stress modulators, or immune modulators warrant targeted investigation. Ionizing radiation can provoke immunogenic cell death and stimulate cyclic GMP-AMP synthase-stimulator of interferon genes (cGAS-STING)-mediated type I interferon production. Preclinical investigations integrating radiation with PARP inhibition have demonstrated enhanced anti-PD-1 effects. If AE-induced complex DNA lesions can similarly elicit immunogenic signaling without considerable systemic genotoxicity, the integration of AE-emitting RLTs with ICIs may convert localized nanometer-scale cytotoxicity into systemic antitumor immunity, particularly in scenarios of minimal residual disease. Preliminary translational trials must incorporate reliable biomarkers, such as neoantigen release, intratumoral type-I IFN signatures, and T-cell clonality, to validate this approach 231, 232.

Nanomedicine and engineered trafficking enhancers are poised to address the delivery barrier that has historically constrained AE-emitting RLTs. Modular nanocarriers incorporating endosomal-escape domains, proton-sponge or membrane-disrupting motifs, nuclear localization sequences, or cleavable linkers can efficiently shuttle payloads from endosomes to the cytosol and then to the nucleus, surpassing passive constructs; proof-of-principle examples include modular nanotransporters and NLS-tagged antibody constructs for nuclear-directed radionuclide delivery. The design challenge is to maximize nucleus-proximal decay events while minimizing reticuloendothelial sequestration and long-term retention in radiosensitive organs. Subject to anatomical and safety considerations, local delivery methods such as intratumoral injection, convection-enhanced administration, or implanted depots can effectively complement systemic techniques 112, 233.

Accurate prediction and treatment planning require nanoscale dosimetry and Monte Carlo-based microdosimetric tools that translate subcellular distributions into expected DNA lesion complexity. Contemporary frameworks, including Geant4-DNA, enable the quantification of single-decay damage spectra concerning emitter-to-DNA distance, chromatin compaction, and chemical stage (radical production), thus allowing for model-driven optimization of linker length, specific activity, and acceptable biodistribution. Transitioning from conceptual design to secure and efficient dosage planning necessitates the integration of these simulations with empirical assessments of subcellular localization, including high-resolution autoradiography or super-resolution imaging of labeled ligands 59.

Recent advances in artificial intelligence (AI)-based structural modeling, such as AlphaFold-3's ability to predict nucleic acid interactions, allow the integration of structural insights into ligand designs targeting DNA or chromatin 234. Computational approaches have also been employed to identify DNA and RNA binding sites and to prioritize grooves or pockets for small-molecule engagement, guiding the placement of radionuclide-linked moieties to maximize proximity to the DNA backbone 235. Methods including nucleic acid-aware docking, scoring models for protein-nucleic acid-ligand complexes, and structure predictors that jointly model proteins, nucleic acids, and ligands offer a framework to select candidates likely to adopt intercalative or groove-binding poses within nanometer distance of phosphodiester backbones. Utilizing molecular dynamics and microscale dosimetry in conjunction with these predictions enables the prioritization of interesting compounds prior to production. This reduces the number of required tests and enhances the possibility of identifying effective DNA-targeted constructs 236, 237.

Translational success will depend on rigorous safety evaluation. First-in-human studies utilizing AE-emitting radionuclides in conjunction with DNA repair inhibitors must incorporate long-term genotoxicity endpoints, such as persistent chromosomal aberrations, micronucleus formation in normal tissues, mutation accumulation assessments, and monitoring for secondary cancers. Standardized nanoscale dosimetry reporting, preclinical models that replicate chromatin organization and cell-cycle variability, and consensus techniques for assessing subcellular radionuclide localization will facilitate regulatory review and expedite the clinical implementation of these technologies.

What is the probability of success for AE-emitting RLTs during the next decade? The optimal strategy for developing clinically applicable AE-emitting RLTs is a systematic, evidence-driven integration process. This signifies the subsequent strategies: (i) employing targeted AE-emitting RLTs (e.g., PARP) in distinctly defined minimal disease contexts with image-guided dosimetry and target binding verification; (ii) integrating these therapies with short-term protocols of DNA damage response modulators under stringent safety oversight and normal tissue genotoxicity evaluation; (iii) utilizing nanoparticles and click chemistry-bioorthogonal conjugation pretargeting methodologies to facilitate the application of short-lived AE-emitting radionuclides while minimizing systemic exposure; and (iv) merging nanoscale dosimetry with AI-driven ligand design to expedite the optimization of lead compounds. The optimal location for initial clinical success is likely where no residual disease or micrometastases exist, as the intracellular accuracy of AE emission is most advantageous in such contexts.

## 7. Conclusions

AE-emitting RLT is an innovative approach that, in conjunction with β- and α-emitting radionuclides, may establish a novel foundation for complementary pillars in theranostics. To date, current limitations and challenges persist that encompass efficient intracellular delivery, precise microdosimetry, and secure implementation in combination therapies. However, recent advancements in click chemistry, bioorthogonal conjugation, nanomedicine, protein engineering, and AI-driven structural modeling offer tangible prospects for surmounting these limitations. Integrating AE-emitting RLT into multimodal treatment approaches could achieve precision oncology by delivering highly localized cytotoxic effects while preserving healthy tissue. Comprehensively, realizing this vision necessitates collaboration among professionals from multiple disciplines, all within a robust translational pipeline.

## Figures and Tables

**Table 1:** Key Characteristics of Clinically Relevant *vs.* Emerging/Experimental AE-emitting Radionuclides. Adapted from 46, 47

	Radionuclide	Half-life	AEs per decay	Average AEs energy per decay (keV)	Average energy per AEs (keV)	CEs per decay	Average CEs energy per decay (keV)	Average energy per CEs (keV)	γ- or β^+^ keV (%)	Production method
Clinical	Copper-64 (^64^Cu)	12.7 h	1.8	2.1	N/P	5.7E-07	N/P	N/P	β^+^, 653(17.9) γ, 1345.8 (0.48)	^64^Ni(p, n)^64^Cu ^68^Zn(p, αn)^64^Cu ^66^Zn(d, α)^64^Cu
	Gallium-67 (^67^Ga)	3.26 d	4.9	6.3-6.6	1.3	0.34	29.7	14.1	γ, 93 (39) γ, 185 (21) γ, 300 (15)	^68^Zn(p,2n)^67^Ga ^67^Zn(p,n)^67^Ga
	Technetium-99m (^99m^Tc)	6.02 h	4.4	0.9	0.2	1.1	15.2	13.8	γ, 140.5 (89)	^99^Mo/^99m^Tc ^100^Mo(p,2n)^99m^Tc
	Indium-111 (^111^In)	2.80 d	14.7	6.9	0.9	0.16	27.9	176.1	γ, 171.3 (90.7) γ, 245.4 (94.1)	^111^Cd(p,n)^111^In
	Iodine-123 (^123^I)	13.27 h	13.7	7.2	0.5	0.16	21.0	222.6	γ, 159 (83)	^123^Te(p,n)^123^I ^124^Xe(n,2n)^123^I
	Iodine-125 (^125^I)	59.40 d	24.9	12.2	0.5	0.94	7.3	7.7	γ, 35 (6.7)	^124^Xe (n,γ) ^125^Xe (β^+^ decay)⇒^125^I
	Terbium-161 (^161^Tb)	6.91 d	10.9	8.94	0.018-50.9	1.4	36.7	26.2	γ, 25.7 (23.2) γ, 48.9 (17.0) γ, 74.6 (10.2)	^160^Gd(n,γ)^161^Gd-→^161^Tb
	Thallium-201 (^201^Tl)	72.91 h	20.9	14.8	0.7	0.91	29.9	32.9	γ, 167 (10) γ, 135 (3)	^203^Tl(p,3n)^201^Pb
Emerging	Cobalt-58m (^58m^Co)	9.04 h	4.2	3.98	N/P	1.0	18.89	N/P	γ, 24.9 (0.04) γ, 810.8 (99.4)	^58^Ni(n,p)^58m^Co ^58^Fe(p,n)^58m^Co ^61^Ni(p,α)^58m^Co
	Germanium-71 (^71^Ge)	11.43 d	5.2	9.2-10.2	N/P	0	198	N/P	γ, 174.9 γ, 708.2	^71^Ga[v,β]^71^Ge ^70^Ge(n,γ)^71^Ge
	Bromine-77 (^77^Br)	2.37 d	6.6	10-15	25	1.7E-02	N/P	N/P	γ, 239 (23.1) γ, 297.2 (4.2) γ, 520.7 (22.4)	^77^Se(p,n)^77^Br ^75^As(^4^He, 2n)^77^Br
	Palladium-103 (^103^Pd)	16.99 d	13.3	8.54	0.034-22.3	1.8E-05	34.97	16.6-39.8	γ, 39.7 (0.07) γ, 357.5 (0.02)	^102^Pd(n,γ)^103^Pd ^103g^Rh(p,n)^103^Pd
	Rhodium-103m (^103m^Rh)	56.11 m	5.8	2.72	0.034-22.3	0.99	34.97	16.6-39.8	γ,39.7(6.0)	^103^Rh(d,d′)^103m^Rh ^103^Rh(α,α′)^103m^Rh
	Cadmium-107 (^107^Cd)	6.50 h	12.4	N/P	N/P	0.95	N/P	N/P	γ, 93.1 (4.7)	^107^Ag(d, 2n)^107^Cd
	Tin-117m (^117m^Sn)	13.80 d	14.2	N/P	N/P	1.15	N/P	N/P	γ, 158.6 (89)	^116^Sn(n,γ)^117m^Sn ^114^Cd(α,n)^117m^Sn
	Antimony-119 (^119^Sb)	38.19 h	23.7	8.9	0.4	0.84	17.0	20.2	γ, 24-29 (89)	^119^Sn(p,n)^119^Sb
	Tellurium-125m (^125m^Te)	57.40 d	22.4	N/P	N/P	1.9	N/P	N/P	γ, 35.5 (25)	^125^Te (*n*,*n*′)^125m^Te
	Cerium-134 (^134^Ce)	3.16 d	N/P	7.2	N/P	N/P	N/P	N/P	β^+^, 1224 γ, 218 (11.4) γ, 440 (25.9)	^139^La(p,6n)^134^Ce
	Lanthanum-135 (^135^La)	18.9 h	12.3	N/P	N/P	2.9E-04	N/P	N/P	γ, 480.5 (1.5) γ, 874.5 (0.16)	^136^Ba(p,2n)^135^La
	Terbium-155 (^155^Tb)	5.32 d	13.9	4.84	38	0.77	N/P	N/P	γ, 86.6 (32.0) γ, 105.3 (25.1)	^155^Gd(d,2n)^155^Tb
	Erbium-165 (^165^Er)	10.36 h	7.2	5.3	N/P	0	N/P	N/P		^166^Er(d,3n)^165^Tm → ^165^Er ^166^Er(p,2n)^165^Tm → ^165^Er ^165^Ho(d,2n)^165^Er
	Platinum-191 (^191^Pt)	2.80 d	13.3	17.8	1.3	0.99	57.1	0.2	γ, 539 (13.7) γ, 409 (8.0) γ, 360 (6.0) γ, 82 (4.9) γ, 172 (3.5)	^190^Pt(n, γ)^191^Pt ^191^Ir(p, n)^191^Pt
	Platinum-193m (^193m^Pt)	4.33 d	27.4	10.9	0.4	2.9	126.8	42.4	γ, 135.5 γ, 149.78	^192^Os(α,3n)^193m^Pt
	Iridium-193m (^193m^Ir)	10.53 d	6.1	N/P	N/P	1	N/P	N/P	γ, 80.2 (0.0045)	^193^Ir(n,n'y)^193m^Ir
	Platinium-195m (^195m^Pt)	4.02 d	36.5	23.1	0.6	2.7	161.4	58.1	γ, 65.1 (22.5) γ. 66.8 (39.0) γ, 75.7 (16.8) γ, 98.9 (11.7)	^194^Pt(n,γ)^195m^Pt ^192^Os(α,n)^195m^Pt
	Mercury-197m (^197m^Hg)	23.8 h	19.4	13.5	037	1.6	203.5	127.0	γ, 134 (33.5) γ, 279 (6.1)	^197^Au(p,n)^197m,g^Hg

**N/P:** Not provided.

**Table 2:** Monte Carlo-based Microdosimetric of Therapeutic Radionuclides

Parameter	β-emitting radionuclides	α-emitting radionuclides	AE-emitting radionuclides
Primary Monte Carlo simulation tool used	TOPAS-nBio, Geant4-DNA	TOPAS-nBio, MCNP6	TOPAS-nBio, Geant4-DNA
Trach Structure	Long, sparse tracks; low ionization density	Straight, dense, High ionization linear tracks	Extremely short tracks (nm scale), dense localized clusters
Linear energy (γ) distribution /LET	< 1 keV/μm	50-200 keV/μm	Extremely localized clusters (per decay absorbed dose 10-100 kGy, within a few nm)
Stochastic variance	High (due to long-range track)	Moderate (predictable Bragg-like)	Extremely high (depending on nuclear/perinuclear localization)
Specific energy delivered to nucleus (per decay)	~10^-4^-10^-3^ Gy • cell^-1^	0.1-1 Gy • cell^-1^	Up to 10-1000 Gy locally within 2-3 nm DNA
Dominant damage type	Mostly SSBs	Dense DSB clusters	Nanometer-scale DSB clusters; based-damage clusters
Cluster DNA damage index	Low (1-2 events/μm track)	High (20-40 events/μm track)	Very high (100-500 events localized per decay)
Effective range	0.2-12 mm	40-80 μm	2-500 nm
Relative biological effectiveness	1-1.3	4-8	8-30 (depends on nuclear localization)
Cellular dose uniformity	Very uniform	High non-uniform	Ultra non-uniform; decay-site dependent
Repair complexity	Low-moderate	High	Highest (multiple DSBs within 10-20 bp)
Microdosimetry concerns	Under-killing at nanoscale	Micrometastases, well-targeted lesions	Precision molecular theranostics; nanometer-scale lethality

**Table 3:** Key Limitations of AE-emitting Radionuclides as Successful RPs: Mechanistic/Technical Basis, Impact on Clinical Translation, and Mitigation Strategies

Limitations	Mechanistic / technical basis	Impact on clinical translation	Potential mitigation strategies
Extreme short range → strong subcellular location dependence	AEs have nm range; lethal effect requires decay at or extremely close to DNA/nucleus	If the radionuclide fails to reach DNA/nucleus, the therapeutic effect is negligible → narrow therapeutic window; high variability between target/cells	Design vectors that intentionally localize to the nucleus (DNA intercalators, nuclear-targeting peptides/proteins, and PARP-targeted agents); validate subcellular distribution quantitatively
Difficulty of achieving reproducible, high fraction of DNA/nucleus uptake	Many targeting vectors (antibodies, peptides) accumulate on the membrane or in the cytosol; only a small fraction reaches nucleus	Low and variable nuclear delivery → inconsistent efficacy across patients/tumor types; hard to power clinical trials	Use small molecules that bind DNA or DNA-associated proteins (PARP inhibitors, nucleoside analogues); engineer endosomal escape and NLS; patient selection by biomarker
Lack of cross-fire for bulky disease	AEs deposit energy over very short distances; they produce little to no cross-fire dose compared with β/α-emitting radionuclides	Ineffective against bulky tumors; narrows clinical indications to microscopic residual disease or disseminated single cells	Target indications where micrometastases or minimal residual disease predominate (adjuvant setting, leptomeningeal disease); combine with agents that debulk tumors
Potential for heterogeneous normal tissue microdosimetry & unexpected toxicity	Small-scale hotspots (microdosimetry) can cause high local doses in normal cells if mis-localized (e.g., kidney, bone marrow microenvironments)	Unanticipated toxicities could appear despite acceptable average organ doses, complicating safety monitoring	Implement microdosimetry risk assessment, a sensitive biomarker of DNA damage in normal tissues, and conservative first-in-human dosing
Radionuclide physical half-life trade-offs	Long half-life isotopes (e.g., [^125^I]) increase non-target exposure; short half-life isotopes (e.g., [^64^Cu]Cu-, [^99m^Tc]Tc-, [^123^I]) require rapid delivery/complex logistics	Logistical factors (production, shipping, and timing) and concerns about non-target doses restrict the range of isotopes feasible for clinical use	Choose an isotope with a half-life matched to the vector PK; optimize the production/supply chain; Use preclinical PK modeling to guide isotope choice
Radiochemistry / *In vivo* stability	Some isotopes have weak chelation chemistry; Radioiodination undergoes *in vivo* dehalogenation	Off-target dose to sensitive organs complicates the safety profile and may limit dosing	Use chelators optimized for AE radionuclides, stabilized metal complexes, metabolism-stabilized linkages, prosthetic groups, or non-iodine AE-emitting radionuclides
Production, supply & radio-pharmacy constraints	Some AE-emitting radionuclides have limited production routes or require on-site cyclotron/complex radiochemistry	Scale-up to multi-center trials and routine clinical use are hindered by supply chain and cost issues	Prioritize clinically scalable isotopes; develop centralized radiopharmacies or generator/cyclotron networks, and ensure regulatory harmonization
Regulatory, trial design & commercialization hurdles	Need for novel microscale dosimetry endpoints, complex manufacturing, and narrow indication complicate approvals and commercial investment	Slow or absent commercial development; difficulty in obtaining funding and executing large trials	Early engagement with regulators; designed phased, biomarker-driven trials; public-private partnership to derisk development
Competition from clinically successful β/α RLTs & limited commercial incentives	β- and α-emitting radionuclides have shown robust clinical success and broader applicability (cross-fire + established dosimetry)	Funding and industry attention tend to favor non-AE approaches, resulting in slower progress for AE methods.	Highlight distinct advantages in niche applications (e.g., high therapeutic index for micrometastases); explore combinations of treatment regimens and seek translational partnerships.

**AE:** Auger electron;** PARP**: Poly(ADP-ribose)polymerase; **NLS**: Nuclear localization sequence; **PK**: Pharmacokinetics

**Table 4:** Nanoparticle-based Radionuclide Carriers for Overcoming the Intrinsic Limitations of AE-emitting RLTs. Adapted from 112.

Category	Types	Radiolabeled strategy	Mechanism / Advantages	Limitations / Challenges	Refs
Organic	MCPs	[^111^In]In- via DTPA chelators on polymer backbone	High specific activity (29 chelators per polymer); amplified radionuclide loading; antibody (e.g., trastuzumab) conjugation for targeting; potential nuclear localization via NLS peptides	Rapid clearance; steric hindrance to receptor binding; polyanionic charge causing non-specific uptake (liver); immunoreactivity; low fraction internalized / slow nuclear transport	113-118
	BCMs	[^111^In]In-DTPA attached to hydrophilic corona	Co-delivery design in which the hydrophobic core carries a radiosensitizer (e.g., methotrexate), surface carries targeting (e.g., trastuzumab Fab) and NLS peptides; enables simultaneous delivery of AE-emitting radionuclide and sensitizer to nucleus; enhances cytotoxicity via synergy	Complex synthesis; potential heterogeneity in micelle formation; limited tumor penetration; PK variability; non-uniform cellular/subcellular distribution; scale-up challenges	119, 120
	Stimuli responsive micelles (PEG-based)	[^125^I] (e.g., on PEG-phenolic compound)	Controlled release occurs in response to stimulus (e.g., laser, pH) to trigger release of [^125^I] near/inside the nucleus; PEG improves circulation time; allows image-guided or light-triggered AE-emitting RLTs	Requires external stimulus (e.g., laser) for activation; tissue penetration of stimulus; complexity of formulation; potential off-target release; stability of radiolabel and formulation *in vivo*	121, 122
	MORF / Streptavidin-based NP	[^111^In]In- via DOTA or NHS-MAG₃ chelators, or [^125^I] labeling on MORF backbone	Modular assembly: streptavidin-biotin enables combination of MORF (oligomer), antibody, NLS, or TAT-peptide; MORF binds RNA / DNA → increases nuclear proximity; high loading; flexible design	Non-specific uptake (liver, kidneys, spleen) *in vivo*; limited internalization/nuclear delivery; immunogenicity (streptavidin); label stability (especially [^125^I], risk of dehalogenation); premature release by weak non-covalent binding	123-126
	Chitosan-based NPs	[^125^I]-labeled antisense oligonucleotide (e.g., antisense AFP) encapsulated in chitosan NP	Gene-targeted therapy: delivery of antisense oligos to reduce target gene expression; positive charge of chitosan enhances cellular uptake; chitosan protects the oligo and brings [^125^I] near DNA / target mRNA; increased DNA damage compared to free oligo	Low *in vivo* delivery efficiency; stability of radiolabeled oligo; cell-type specificity concerns; potential toxicity of chitosan NPs; not fully addressed with clearance and biodistribution	127
	Dendrimers (e.g., PAMAM)	[^111^In]In- via many DTPA or DOTA chelators on peripheral amines	Very high payload (many chelators per dendrimer); well-defined, monodisperse structure; multivalency allows for attachment of targeting ligands and possibly drugs; cationic surface promotes cell uptake via electrostatics; high internalization (~77.6% in SHIN-3 cells after 24 h in one study)	Biodistribution issues: long-term accumulation (liver, kidney) for higher-generation dendrimers; potential toxicity due to cationic surface; radiolabel stability; slow clearance; synthetic complexity; GMP translation challenges	128-131
	Liposomes	[^125^I]-daunorubicin derivative encapsulated; or [^111^In]In-labeled peptide (e.g., hEGF) loaded and chelated	High loading capacity; PEGylation improves circulation; surface functionalization (antibody, ligand) for targeting; two-step strategies: e.g., internalization, then release, then nuclear delivery; controlled release (e.g., ultrasound-triggered cavitation)	Penetration in solid tumors is limited; RES clearance; possible leakage of cargo; triggered release (e.g., ultrasound) may not be efficient *in vivo*; radiolabel release, *in vivo* stability; lack of comprehensive *in vivo* toxicity/efficacy data	132-134
Inorganic	Gold NPs (AuNPs)	[^125^I] directly bound to the gold surface; or [^111^In]In- via chelator (DTPA / DOTA) on surface-modified AuNPs	High surface area allows multivalent ligand attachment for targeting; PEGylation for stability; perinuclear accumulation observed; high-Z potential for secondary electron production (photo, AE) when combined with external radiation; dual-modality theranostics possible	RES uptake and rapid clearance; limited tumor accumulation after IV Injection; steric hindrance reduces targeting affinity; long-term retention, potential toxicity; surface modification complexity; *in vivo* translation challenges; low IV tumor uptake (1.2% IA/g in one study); intratumoral injection needed for high uptake	135-139
	Platinum NPs / Core-shell Platinum structures	[^193m^Pt]Pt, [^195m^Pt]Pt (intrinsic radionuclide)	Enhanced high-Z platinum via conversion electrons; core-shell design allows control of surface chemistry and stability; potential to combine with chemotherapy (Pt-based)	Limited data in the context of AE-emitting RLTs; complex synthesis; radiolabel stability; toxicity concerns; unknown biodistribution/clearance; scale-up and regulatory hurdles	140
	Titanium dioxide (TiO₂) NPs	[^125^I] attached to surface (halogen)	High surface reactivity; inherent stability; potential for radical generation (e.g., •OH) after decay/activation; high-Z enhancement of local radiation dose; surface functionalization possible	Limited *in vivo* data; radiolabel instability (I-dehalogenation risk); long-term biocompatibility / toxicity unclear; clearance and biodegradation not well established; possible oxidative damage to non-target tissues	141
Inorganic / Coordination Polymer (High-Z)	High-Z core (Hf) porphyrin coordination polymer NPs	Using Hf and porphyrin ligand; potential for radionuclide attachment	Hf allows dose amplification by external radiation (photoelectron cascade); porphyrin offers multifunctionality (imaging, targeting, possible photosensitizer); a biodegradable coordination structure; potential for tumor accumulation via EPR	Limited specific examples in the context of AE-emitting radionuclides; synthesis complexity; radiolabel stability; immunogenicity; unknown PK; lack of *in vivo* therapy data; scale-up to clinical grade challenging	142

**MCP**: Metal-chelating polymer; **DTPA**: Diethylenetriaminepentaacetic acid; **NLS**: Nuclear localization sequence; **BCM**: Block copolymer micelle; **PEG**: Polyethylene glycol; **MORF**: morpholino oligomer; **NP**: Nanoparticle; **DOTA**: 1,4,7,10-tetraazacyclododecane-1,4,7,10-tetraacetic acid; **NHS-MAG_3_**: 1,4,7,10-tetraazacyclododecane-1,4,7,10-tetraacetic acid; **TAT**: Trans-activating transcriptional activator; **AFP**: Alpha-fetoprotein; **PAMAM**: poly(amidoamine); **GMP**: Good manufacturing practices; **hEGF**: Human epithelial growth factor; **RES**: Reticuloendothelial system; **IV**: Intravenous; **% IA/g**: Percentage of injected activity per grams **EPR**: Enhanced permeability and retention

**Table 5:** Historical Use and Prior Development on AE-emitting RLTs

Radionuclide	Agent	Target	Tumor (or model)	Primary findings	Refs
[^125^/^123^I]	[^125^I]IUdR	Thymidine of DNA	Hamster Chinese Lung Fibroblast (V79)	The potent high-LET cytotoxicity of [^125^I]IUdR was demonstrated by its ability to induce significantly more DNA DSBs and cell death at only 0.0037 Bq per cell.	157
			Rat Leptomeningeal metastases (9L)	The use of [^125^I]IUdR effectively prolonged the time to paralysis and showed selective retention in tumor and thyroid tissues, which indicates targeted antitumor activity.	158
			Patient with liver metastases from colorectal cancer	Given the sustained uptake of [^125^I]IUdR in liver tumors and the limited fraction of S-phase cells at any given time (15-50%), repeated intra-arterial injections are necessary to achieve effective tumor cell inactivation.	159
	[^123^I]IUdR		Murine ovarian tumor (MOT) cells originated spontaneously in C3H female mice	Selective uptake of [^123^I]IUdR in tumor-bearing models with 1% of injected administered activity associated with MOT cells 24 h post-injection significantly prolonged survival, increasing median survival by 11 d and achieving 20% absolute survival at the highest administered activity.	160
			Patient with liver metastases from colorectal cancer	Biochemical modulation with 5-FU and folinic acid increased early tumor uptake of [^123^I]IUdR from 9.1% to 14.9% IA, representing a 72% enhancement that remained stable up to 42 h post-infusion.	161
	[^125^I]DCIBzL	PSMA	Human prostate cancer cell line with PSMA-positive (PC3-PIP)	[^125^I]DCIBzL selectively induced DNA damage and suppressed clonogenic survival in PC3-PIP cells, leading to significantly delayed tumor growth *in vivo* compared with PC3-Flu controls.	162
			Micrometastatic prostate cancer cell line derived from metastatic lumbar vertebrae (PC3-ML)	Treatment of [^125^I]DCIBzL at therapeutic administered activity (≥ 18.5 MBq) delayed metastasis, improved median survival, and exhibited minimal toxicity, with dosimetric modeling supporting a favorable therapeutic window due to low renal nuclear administered activity relative to tumor cell nuclei.	163
	[^125^I]CLR1404	APC	Triple-negative breast cancer (TNBC, MDA-MB-231)	[^125^I]CLR1404 demonstrated favorable tumor-to-bone marrow dosimetry and was well tolerated at a therapeutically administered activity (74 MBq), producing approximately a 60% reduction in TNBC tumor volume, delaying metastatic progression, and significantly extending survival in TNBC models.	164
	[^125^I]35A7	CEA	Peritoneal carcinomatosis (A431)	Targeting CEA with non-internalizing [^125^I]35A7 resulted in enhanced tumor control and survival compared with [^125^I]m225, owing to greater tumor retention and reduced catabolite loss, demonstrating that efficient AE-emitting RLTs can be achieved without the need for nuclear targeting.	165
			Peritoneal carcinomatosis (A431)	The promising therapeutic index of short-course intraperitoneal [^125^I]35A7, characterized by high tumor targeting and low off-target toxicity, supports its integration with radiation-enhancing drugs in the post-surgical management of small-volume peritoneal disease.	166
			Human colorectal cancer (p53^+/+^/or p53^-/-^HCT-116)	The accumulation of DNA DSBs and the resulting micronuclei formation following exposure to [^125^I]35A7, regardless of internalization, indicates that hypersensitivity arises from defective DNA repair mechanisms at low administered activity rates.	167
	[^125^I]CO17-1A	EpCAM	Human colon carcinoma (GW-39)	[^125^I]CO17-1A exhibits superior tumor suppression compared to [^131^I]CO17-1A despite similar toxicity profiles, suggesting that therapeutic efficacy may be influenced more by radionuclide characteristics than by antibody internalization.	168
	[^125^I]mAb-425	EGFR	Patient with GBM and AAF	In a Phase I/II trial with 180 patients who had high-grade gliomas, adding [^125^I]mAb-425 to their treatment significantly increased their chances of living longer, especially for patients under 40 with high Karnofsky scores. This supports its possible use in treating GBM and AAF.	21, 169, 170
[^111^In]In-	[^111^In]In-CO17-1A	EpCAM	Human colon carcinoma (GW-39)	[^111^In]In-CO17-1A provided greater therapeutic efficacy than its [^90^Y] counterparts at matched toxicity levels, indicating the possible use of AE-emitting radionuclides in targeted radioimmunotherapy.	168
	[^111^In]In-DTPA^0^-Octreotide	SSTR-2	Patient with malignant NET	[^111^In]In-DTPA^0^-octreotide treatment in patients with advanced NETs showed minimal toxicity and induced disease stabilization or tumor shrinkage in a substantial subset, particularly among those with higher tumor radioligand accumulation.	171
			Various carcinomas with SSTR-2 positivity	While [^111^In]In-DTPA^0^-octreotide provided clinical benefit with preserved renal function due to the limited range of AEs, cumulative administered activities above 100 GBq posed a risk of hematologic complications, such as myelodysplastic syndrome.	18
			Neuroendocrine liver metastases	Although intra-arterial administration of [^111^In]In-DTPA^0^-octreotide in patients with hepatic NETs showed favorable tumor responses and a median overall survival of 32 months, subsequent studies revealed limited long-term efficacy and raised safety concerns due to γ-emission.	20
	[^111^In]In-DTPA-hEGF	EGFR	Human breast cancer cell with EGFR positive (MDA-MB-468)	With high nuclear uptake and up to 25 Gy delivered per cell, [^111^In]In-DTPA-hEGF effectively reduced the viability of MDA-MB-468 cells and showed no hepatotoxicity or nephrotoxicity *in vivo*, highlighting its promise as a targeted therapy for hormone-resistant breast cancer.	172
				[^111^In]In-DTPA-hEGF induced tumor regression in MDA-MB-468 xenografts (slopes: 0.009 and 0.0297 d^-1^, P < 0.001) and delivered up to 1400 cGy to the cell nucleus, supporting its use for micrometastatic breast cancer.	173
				[^111^In]In-DTPA-hEGF induced nuclear translocation (131 ± 6 MBq/nucleus) and significant DNA damage (35 ± 15 γ-H2AX foci) in MDA-MB-468 cells, resulting in a surviving fraction of 0.013 ± 0.001, which correlated with EGFR expression.	174
			Patients with metastatic breast cancer (EGFR-positive)	[^111^In]In-DTPA-hEGF demonstrated a favorable safety profile in a Phase I trial, with no administered activity-limiting toxicities up to 2290 MBq, rapid blood clearance, low administered activity to normal organs, and visible tumor accumulation in 47% of patients, although no objective tumor responses were observed.	22
	[^111^In]In-DTPA-NLS-Trastuzumab	HER2	Human breast cancer cell lines with HER2 positive (SK-BR-3, MDA-MB-361)	Conjugation of [^111^In]In-DTPA-Trastuzumab with 6 NLS peptides enhanced nuclear localization in HER2-positive breast cancer cells (e.g., internalization in SK-BR-3 increased from 7.2% to 14.4%, and nuclear uptake in xenografts from 1.1% to 2.4-2.9%), resulting in up to a 6-fold increase in cytotoxicity compared with unlabeled trastuzumab and a 5-fold increase compared with [^111^In]In-DTPA-Trastuzumab.	115
	[^111^In]In-NLS-HuM195	CD33	Human leukemia cell line (HL-60)	[^111^In]In-NLS-HuM195 achieved potent AML cell killing by increasing nuclear uptake up to 66% and reducing IC_50_ and IC_90_ values by over 50% compared to non-NLS controls, eliminating HL-60 colonies at 3.33 MBq/cell and showing no adverse effects *in vivo*, highlighting its therapeutic potential.	175
			Mitoxantrone-resistant HL-60 cell line (HL-60-MX-1)	[^111^In]In-NLS-Trastuzumab significantly enhanced nuclear uptake and cytotoxicity against HL-60-MX-1 cells, with patient-derived AML specimens also showing variable but positive responses, suggesting efficacy against MDR phenotypes, including Pgp-170, BCRP1, and MRP1.	176
[^161^Tb]Tb-	[^161^Tb]Tb-DOTATOC	SSTR-2/5	Patient with paraganglioma (metastatic, well-differentiated, nonfunctional malignant) and neuroendocrine neoplasm of pancreas tail (metastatic, functional)	A first-in-human study demonstrated that [^161^Tb]Tb-DOTATOC, synthesized with high radiochemical purity, enabled high-quality SPECT/CT imaging and detection of small bone and liver metastases at low administered activities, showing favorable biodistribution in the liver, kidneys, spleen, and bladder without any reported adverse effects.	177
	[^161^Tb]Tb-DOTATATE	SSTR-2	Patients with NET (SSTR positive)	Substitution of [^177^Lu]Lu- with [^161^Tb]Tb-DOTATATE Therapy boosts tumor absorbed dose per administered activity by approximately 40% (e.g., 2.9 → 4.1 Gy/GBq for a 10 g tumor), but to avoid increased kidney and bone marrow toxicity, the standard 7.4 GBq administered activity should be reduced to 5.3-5.4 GBq of [^161^Tb]Tb-DOTATATE.	178
	[^161^Tb]Tb-DOTA-cm09	FR	Human nasopharyngeal/ovarian cancer cell line (KB/IGROV-1) with FR-positive	[^161^Tb]Tb-DOTA-cm09 showed superior therapeutic efficacy than [^177^Lu]Lu-DOTA-cm09 both *in vitro* and *in vivo*, requiring significantly lower IC_50_ values in FR-positive tumor cells and delivering a higher tumor dose per administered activity (3.3 Gy/MBq *vs*. 2.4 Gy/MBq), while maintaining imaging capabilities and renal safety over a 6-month observation period.	179
	[^161^Tb]Tb-SibuDAB	PSMA (High affinity with albumin)	Human prostate cancer with PSMA-positive (PC3-PIP)	Compared to [^177^Lu]Lu- counterparts, [^161^Tb]Tb-SibuDAB and PSMA-I&T exhibited similar biodistribution but provided ~ 40% higher tumor-administered activities, with [^161^Tb]Tb-SibuDAB showing markedly enhanced tumor uptake (up to 69% IA/g) and therapeutic efficacy without observable toxicity in mice.	180
			Patients with mCRPC	[^161^Tb]Tb-SibuDAB achieved superior tumor retention and absorbed dose per administered activity delivery (6.5 Gy/GBq, T_h_ = 135 h) compared with [^177^Lu]Lu-PSMA-I&T (2.6 Gy/GBq, T_h_ = 67 h) in the first mCRPC patient, with no acute toxicity despite modestly higher kidney (2.6 *vs*. 1.2 Gy/GBq) and parotid (0.5 *vs*. 0.3 Gy/GBq) absorbed doses administered activities (PROGNOSTIC Phase I clinical trial, NCT06343038).	181
	[^161^Tb]Tb-PSMA-617	PSMA	6 patients with mCRPC	[^161^Tb]Tb-PSMA-617 showed superior efficacy in mCRPC patients, with a 2.4-fold increase in tumor absorbed dose per administered activity (6.10 ± 6.59 *vs*. 2.59 ± 3.30 Gy/GBq) and higher therapeutic indices for the kidneys (11.54 ± 9.74 *vs*. 5.28 ± 5.13 Gy/GBq) and parotid glands (16.77 ± 13.10 *vs*. 12.51 ± 18.09 Gy/GBq) (NCT04833517).	182
	[^161^Tb]Tb-DOTA-LM3	SSTR-2	Rat pancreas tumor cell line with SSTR-positive (AR42J)	Dual-isotope SPECT/CT imaging in AR42J tumor-bearing mice demonstrated that [^161^Tb]Tb- and [^177^Lu]Lu-labeled somatostatin analogues (DOTATOC and DOTA-LM3) exhibited indistinguishable PK and sub-organ biodistribution, with DOTA-LM3 showing significantly higher tumor uptake than DOTATOC (e.g., > 20% IA/g *vs*. ~10% IA/g at 4 h post-injection).	183
			Patient with ileal NET (metastatic, hormone-active [carcinoid syndrome])	Following administration of 1 GBq [^161^Tb]Tb-DOTA-LM3, the patient's liver metastases demonstrated a tumor half-life of 130 h and an absorbed dose per administered activity of up to 39 Gy/GBq, while bone marrow, kidney, and spleen absorbed doses per administered activity were 0.31, 3.33, and 6.86 Gy/GBq, respectively, accompanied by a chromogranin A decrease of 163 µg/L and minimal hematologic toxicity (NCT05359146).	184

**MOT**: Murine ovarian tumor; **IA**: Injected activity; **APC**: Alkylphophosphocoline; **TNBC**: Triple-Negative breast cancer; **CEA**: Carcinoembryonic antigen; **EpCAM**: Epithelial cell adhesion molecule; **EGFR**: Epidermal growth factor receptor; **GBM**: Glioblastoma multiforme; **AAF**: Astrocyte with anaplastic foci; **NET**: Neuroendocrine tumor; **NLS**: Nuclear localization sequence; **AML**: Acute myeloid leukemia; **DTPA**: Diethylenetriaminepentaacetic acid; **HER2**: Human epidermal growth factor receptor 2; **FR**: Folate receptor; **IC_50_**: concentration required to inhibit cell growth by 50%; **SSTR**: Somatostatin receptor; **mCRPC**: metastatic castration-resistant prostate cancer.

## Data Availability

The data analyzed and summarized is included within the manuscript.

## Abbreviations

RLT: Radionuclide therapy
TRT: Targeted radionuclide therapy
RP: Radiopharmaceuticals
AE: Auger electrons
EBRT: External beam radiotherapy
ICIs: Immune checkpoint inhibitors
FDA: Food and drug administration
GEP-NET: Gastroenteropancreatic-neuroendocrine tumor
PSMA: Prostate specific membrane antigen
mCRPC: metastatic castration-resistant prostate cancer
TPR: Tumor penetration range
LET: Linear energy transfer
DNA SSB/DSB: DNA single-strand breaks/double-strand breaks
SPECT: Single photon emission computed tomography
PSA: prostate-specific antigen
EC: Electron capture
IC: Internal conversion
TEPC: Tissue-equivalent proportional counter
RBE: Relative biological effectiveness
CE: Conversion electron
P/E: Photon-to-electron
DOTA: 1,4,7,10-tetraazacyclododecane-1,4,7,10-tetraacetic acid
DTPA: Diethylenetriaminepentaacetic acid
NOTA: 1,4,7-triazacyclononane-1,4,7-triacetic acid
EDTA: Ethylenediaminetetraacetic acid
PARP: Poly(ADP ribose) polymerase
NLS: Nuclear localization sequence
GBM: Glioblastoma multiforme
TNBC: Triple-negative breast cancer
PK: Pharmacokinetics
NP: Nanoparticle
MCP: Metal-chelating polymer
BCM: Block copolymer micelle
PEG: Polyethylene glycol
MORF: morpholino oligomer
NP: Nanoparticle
NHS-MAG3: 1,4,7,10-tetraazacyclododecane-1,4,7,10-tetraacetic acid
TAT: Trans-activating transcriptional activator
AFP: Alpha-fetoprotein
PAMAM: poly(amidoamine)
GMP: Good manufacturing practices
hEGF: Human epithelial growth factor
RES: Reticuloendothelial system
% IA/g: Percentage of injected activity per gram
EPR: Enhanced permeability and retention
SPAAC: Strain-promoted azide-alkyne cycloaddition
IEDDA: Inverse electron-demand Diels-Alder
TBR: Tumor-to-background ratio
PET: Positron emission tomography
ABD: albumin-binding domains
TME: Tumor microenvironment
MOT: Murine ovarian tumor
IA: Injected activity
APC: Alkylphophosphocoline
CEA: Carcinoembryonic antigen
EpCAM: Epithelial cell adhesion molecule
EGF: Epidermal growth factor
EGFR: Epidermal growth factor receptor
AAF: Astrocyte with anaplastic foci
NET: Neuroendocrine tumor
AML: Acute myeloid leukemia
HER2: Human Epidermal growth factor Receptor 2
FR: Folate receptor
IC_50/90_: Concentration required to inhibit cell growth by 50%/90%
SSTR: Somatostatin receptor
IUdR: 5-Iodo-2′-deoxyuridine
[^125^I]DCIBzL: 2-[3-[1-carboxy-5-(4-[^125^I]iodo-benzoylamino)-pentyl]-ureido]-pentanedioic acid
MIP: Maximum-intensity-projection
ROIs: Region of interests
RIT: Radioimmunotherapy
IV: Intravenous
Bip: brief intraperitoneal
MTA: Maximum tolerated activity
MTD: Maximum tolerated dose
cGAS-STING: cyclic GMP-AMP synthase-stimulator of interferon genes
MACROPA: 6-[[16-[(6-carboxypyridin-2-yl)methyl]-1,4,10,13-tetraoxa-7,16-diazacyclooctadec-7-yl]methyl]pyridine-2-carboxylic acid
AI: Artificial intelligence
